# The pH-dependence of efflux ratios determined with bidirectional transport assays across cellular monolayers

**DOI:** 10.1016/j.ijpx.2024.100269

**Published:** 2024-07-08

**Authors:** Soné Kotze, Kai-Uwe Goss, Andrea Ebert

**Affiliations:** a Department of Computational Biology and Chemistry, Helmholtz Centre for Environmental Research (UFZ), Permoserstraße 15, Leipzig 04318, Germany; b Institute of Chemistry, University of Halle-Wittenberg, Kurt-Mothes-Straße 2, Halle 06120, Germany

**Keywords:** Active transport, Efflux, P-glycoprotein, Transporters, MDCK assays, Efflux ratio

## Abstract

MDCK/Caco-2 assays serve as essential in vitro tools for evaluating membrane permeability and active transport, especially mediated by P-glycoprotein (P-gp). Despite their utility, challenges remain in quantifying active transport and using the efflux ratio (ER) to determine intrinsic values for active efflux. Such an intrinsic value for P-gp facilitated efflux necessitates knowing whether this transporter transports the neutral or ionic species of a compound. Utilising MDCK-MDR1 assays, we investigate a method for determining transporter substrate fraction preference by studying ER pH-dependence for basic, acidic and non-dissociating compounds. These results are compared with model fits based on various assumptions of transporter species preference. As an unexpected consequence of these assays, we also give evidence for an additional influx transporter at the basolateral membrane, and further extend our model to incorporate this transport. The combined influences of paracellular transport, the previously unaccounted for basolateral influx transporter, as well as potential pH effects on the transporter impedes the extraction of intrinsic values for active transport from the ER. Furthermore, we determined that using inhibitor affects the measurement of paracellular transport. While clear indications of transporter species preference remain elusive, this study enhances understanding of the MDCK system.

## Graphical abstract

**Figure:**
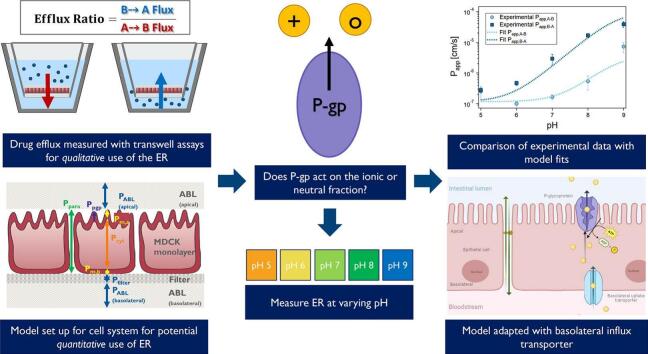

## 1 Background

Bidirectional MDCK or Caco-2 assays are the in vitro gold-standards for investigating the epithelial permeability and intestinal absorption of compounds, as well as the involvement of active transport in these processes (Wang and Sauerwald, 2014; Bohets et al., 2001; Volpe, 2011). MDCK cells transfected with the ABCB1 gene over-express the efflux transporter P-glycoporotein (P-gp), also known as multidrug resistance protein 1 (MDR1) (Pastan et al., 1988). P-gp is one of the most important membrane proteins, as its broad substrate specificity and efflux activity is responsible for actively pumping out xenobiotics from cells, thereby reducing the efficacy of drug delivery for several important pharmaceuticals (Juvale et al., 2022; Sharom, 2008; Kim, 2002). MDCK-MDR1 assays are thus recommended by regulatory agencies during drug development to evaluate any potential role P-gp may play in the uptake and distribution of pharmaceuticals, as well as any potential drug-drug interactions (FDA, 2020; FDA, 2022; EMA, 2012). However, these assays are not limited to P-gp, as the activity of any efflux transporter can be evaluated provided that a suitable cell line is used (e.g transfected with the efflux pump of interest, or knocking-out the genes of other prominent transporters (Gallagher et al., 2016; Gartzke et al., 2015; Wegler et al., 2021)), or transporter-specific inhibitors are used to evaluate the contribution of the pump of interest towards efflux activity. It is often recommended to determine whether drugs are substrates of the two most consequent transporters: P-gp and breast cancer resistance protein (BCRP). This can be determined by measuring the transport (i.e apparent permeability, $P_{\mathrm{app}}$) of a given compound between a donor and acceptor compartment, separated by the cellular monolayer grown on a permeable support (filter). For bidirectional assays, flux (J) is measured in both the apical-to-basolateral $\left(A\to B\right)$ direction and the basolateral-to-apical direction $\left(B\to A\right)$ and the quotient of these values results in the widely-used metric to evaluate active efflux known as the efflux ratio (ER):

(1) $$\mathrm{ER}\equiv \frac{J_{B\to A}}{J_{A\to B}}$$

The ER is primarily used as a qualitative metric, and often establishing that it is greater than a threshold value (usually 2) suffices to classify a compound as a substrate of the investigated transporter (FDA, 2020). However, efforts have also been made to derive a relationship which would allow the quantification of active transport based on the ER (Sugano et al., 2011), which in itself cannot meaningfully be used as a measure for active transport as it is merely an indication of the interplay between passive and active transport. The following relationship was derived which expresses the ER as a function of active efflux facilitated by a transporter $\left(P_{\mathrm{pgp}}\right)$, and the passive permeability through the apical membrane $\left(P_{m,a}\right)$ which runs parallel to the transporter:

(2) $$\mathrm{ER}=\frac{P_{\mathrm{pgp}}}{P_{m,a}}+1$$

From Eq. (2) it is clear that this simple relationship has the potential to be very meaningful, as it provides direct access to a proposed intrinsic value for active transport, $P_{\mathrm{pgp}}$, if the ER and the passive membrane permeability of the compound are known. This intrinsic value for active transport, analogous to intrinsic membrane permeability $\left(P_{0}\right)$ for passive transport, would allow a quantitative assessment of a compound's efflux which would be far more consequential than the ER. In a recent publication we proved that the fundamental ER relationship to active efflux and apical membrane permeability is not affected by the filter or aqueous boundary layers (ABL), even though these resistances may greatly affect individual $P_{\mathrm{app}}$ values (Kotze et al., 2024). We also showed that when paracellular transport (the movement of molecules through the tight junctions between cells (Bittermann and Goss, 2017)) dominates the flux in both directions, the ER is reduced to unity, thereby masking any potential efflux. Thus, we conclusively established that great care must be taken to avoid paracellular dominance in these transport assays, whereas measures to avoid ABL or filter limitation are unnecessary. Furthermore, any influence of paracellular transport (even when it is not dominant) on the ER must be accounted for in the model. Therefore, Eq. (2) in this form is only valid in very specific cases, and we derived a more encompassing version that accounts for paracellular transport and speciation.

In this study we further probed this relationship and its potential for enabling the determination of intrinsic values for active efflux. Our focus pivots to ionisable compounds, and thus speciation and fractionation factors become relevant in the mathematical equations. It thus becomes essential to know which species the efflux transporter preferentially acts on. As such, our aims were i) to explore a potential method for determining the efflux pump's substrate fraction preference. We investigate this using P-gp, and our evaluation is based on data from bidirectional MDCK-MDR1 assays performed across a range of pH values for acidic, basic and non-dissociating chemicals. These assays revealed a more complex system than anticipated. Notably, we obtained evidence for the presence of an additional influx transporter in the basolateral membrane which has to be considered in the model for any meaningful interpretation of the MDCK-MDR1 data. This unspecified transporter, usually not considered in the evaluation of transport experiments, has already been proposed by Troutman and Thakker (2003). Therefore, our second aim was to extend our model to encompass transport across the basolateral membrane facilitated by an additional influx transporter, and include it in our analyses of transporter species preference. Consequently, the pH-dependence data are compared with model fits based on different assumptions: a) Both transporters preferentially act on the neutral species, b) both transporters preferentially act on the ionic species and c) the transporters have different species preferences.

## 2 Theory

### 2.1 Multi-barrier model

The base model used in our analysis was set up to predict the passive permeability of organic chemicals through cellular monolayers (Bittermann and Goss, 2017). It was later adapted to account for the so-called concentration-shift effects described by Dahley et al. (2023) that occur when there is a pH difference between the apical and basolateral compartment. Finally, in our previous study, we further modified the model to include efflux from the apical membrane (Kotze et al., 2024). A detailed look at the model can be found there, but a brief description follows.

In bidirectional transwell assays, chemicals are transported between the apical and basolateral compartments, and the permeability is measured in both the A→B direction and B→A direction. As the chemical passes from one compartment to another through the cellular monolayer (known as the transcellular pathway), it encounters several permeation barriers: The apical ABL (ABL,a), followed by the apical membrane of the cell (m,a) in which P-gp is embedded (pgp), the cytosol (cyt), the basolateral membrane (m,b) in which the proposed influx transporter is embedded (b), the filter and finally, the basolateral ABL (ABL,b). However, chemicals can also take the paracellular route and instead diffuse through the water-filled pores found in tight junctions between cells.

**Fig. 1:**
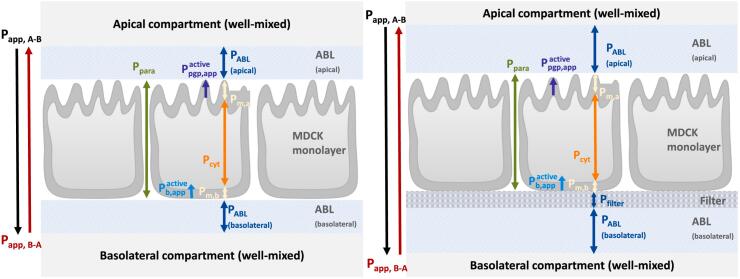
Permeation barriers and associated permeabilities in vivo and in vitro. In vivo, well-mixed donor and acceptor compartments are separated by apical ABL, cell monolayer, and basolateral ABL. The in vitro system introduces a filter layer and thicker ABL (not to scale). Adapted from Dahley et al. (2023) and Kotze et al. (2024) to include active efflux facilitated by P-gp $\left(P_{\mathrm{pgp}}\right)$ and cellular influx facilitated by a basolateral transporter $\left(P_{b}\right)$.

The total measured $P_{\mathrm{app}}$ is comprised of the contributions of all these individual permeabilities found in series and parallel. Fig. 1 shows how measured $P_{\mathrm{app}}$ can be sub-divided into its constituent parts, which allows for their individual evaluation. The contribution of each sub-process, if fully understood, can be quantified. Fig. 1 also highlights the important differences between the in vitro transwell setup and the simplified depiction of the in vivo scenario: the presence of the filter and the increased thickness of the ABLs in vitro.

**Fig. 2:**
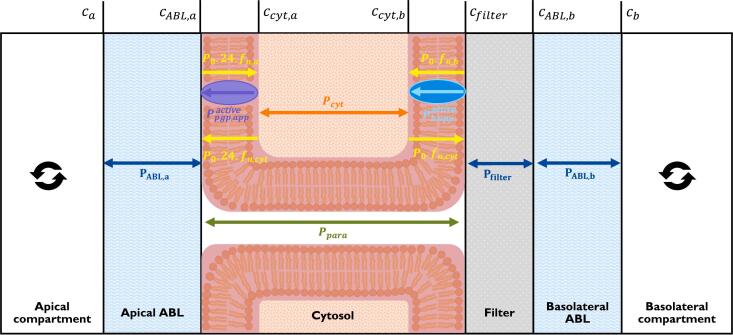
Permeation barriers, associated permeabilities and concentrations in Caco-2 and MDCK transwell assays. Adapted from Dahley et al. (2023) to include active efflux facilitated by P-gp, and from Kotze et al. (2024) to include a basolateral influx transporter. The well-mixed donor and acceptor compartments are separated by apical ABL, cell monolayer, filter and basolateral ABL. All concentrations shown are freely-dissolved aqueous concentrations in the indicated compartment.

Fig. 2 depicts the compartments and permeation resistances that characterise our model. Crucial to note once again is that active transport (in this case $P_{\mathrm{pgp}}^{\mathrm{active}}$ and $P_{b}^{\mathrm{active}}$) differs from passive transport in two important ways: it is unidirectional, and it is not driven by a concentration gradient, but by the substrate concentration at the transporter's binding site. The total permeability of all the barriers depicted in Fig. 2 is determined from the multiple serial and parallel resistances. The two different routes the chemical may use to cross the monolayer (para and trans) are found in parallel with one another. The ABLs and filter are serial resistances. Within the trans pathway, the apical membrane, the cytosol, and the basolateral membrane are connected in series, while active transport is always in parallel to the passive diffusion of the membrane in which it is embedded.

According to the pH-partition hypothesis, it is well-established that only the neutral fraction $\left(f_{n}\right)$ of a chemical can pass through membranes because of its hydrophobic nature (Shore et al., 1957). As such, the intrinsic permeability of the membrane is multiplied with the $f_{n}$ to obtain the pH-dependent membrane permeability:

(3) $$P_{m}=P_{0}\cdot f_{n}$$

Because the apical membrane is folded to form tiny microvilli, it has an increased surface area compared to that of the basolateral membrane. As a result, a factor of 24 is included to calculate apical membrane permeability (Palay and Karlin, 1959) (see Supplementary Material for definitions of all individual permeabilities, as well as calculations of ionic fractions). However, this factor is uncertain due to differences between Caco-2 cells and MDCK cells (Meng et al., 2017), and it has been reported as being lower for MDCK cells (Butor and Davoust, 1992). As such, the use of this factor may lead to an underestimation of the resistance of the apical membrane.

### 2.2 Model adaptations

In this study, two important changes were made to the previously published model. The first being the inclusion of the aforementioned influx transporter in the basolateral membrane, the permeability of which we denote as $P_{b}^{\mathrm{active}}$ (this will be discussed in detail in Section 4.2). The second adaptation stems from the conjecture that the transporters may favour a certain species of an ionisable chemical, in which case the $f_{n}$ or $f_{i}$ (ionic fraction) would need to be factored into their permeabilities. As such, we define the apparent permeability facilitated by P-gp and the basolateral transporter as $P_{\mathrm{pgp},\mathrm{app}}^{\mathrm{active}}$ and $P_{b,\mathrm{app}}^{\mathrm{active}}$, respectively. These variables are defined as follows under the different scenarios:

1. The transporter preferentially acts on the neutral species

(4) $$P_{\mathrm{pgp},\mathrm{app}}^{\mathrm{active}}=P_{\mathrm{pgp}}\cdot f_{n,\mathrm{cyt}}$$

2. The transporter preferentially acts on the ionic species

(5) $$P_{\mathrm{pgp},\mathrm{app}}^{\mathrm{active}}=P_{\mathrm{pgp}}\cdot f_{i,\mathrm{cyt}}$$

For P-gp, as an efflux transporter which pumps molecules out of the cell, the speciation factors are applied to the cytosolic concentration (4–5). These equations are explicitly depicted for P-gp, but the same principles apply for the treatment of the basolateral transporter. In the scenario where basolateral influx transport is included, and active transport permeabilities are expressed as apparent permeabilities to account for potential speciation preferences (and assuming the iso-pH method where the basolateral and apical pH is the same), the following expression is mathematically derived for the absorptive steady-state flux under infinite sink conditions $\left(C_{b}=0\right)$:

(6) $$J_{A\to B}=\frac{1}{\frac{1}{P_{\mathrm{ABL},a}}+\frac{1}{P_{\mathrm{trans},A\to B}+P_{para}}+\left(\frac{P_{trans,B\to A}+P_{para}}{P_{trans,A\to B}+P_{para}}\right)\cdot \left(\frac{1}{P_{\mathrm{filter}}}+\frac{1}{P_{\mathrm{ABL},b}}\right)}\cdot C_{a}$$

where:

(7) $$P_{\mathrm{trans},A\to B}=\frac{1}{\frac{1}{P_{0}\cdot 24\cdot f_{n,a}}+\left(1+\frac{P_{\mathrm{pgp},\mathrm{app}}^{\mathrm{active}}}{P_{0}\cdot 24\cdot f_{n,\mathrm{cyt}}}\right)\cdot \left(\frac{1}{P_{\mathrm{cyt}}\cdot \frac{f_{n,a}}{f_{n,\mathrm{cyt}}}}+\frac{1}{P_{0}\cdot f_{n,a}}\right)}$$

The accompanying equations for overall secretive flux $J_{B\to A}$ and $P_{\mathrm{trans},B\to A}$, as well as full derivations of flux equations and $P_{\mathrm{trans}}$ in both directions can be found in the Supplementary Material. Derivations without a basolateral transporter can be found in Kotze et al. (2024). Substituting the flux equations into Eq. (1) (iso-pH method) results in the following expression for the ER:

(8) $$\mathrm{ER}=\frac{P_{\mathrm{trans},A\to B}\cdot \left(1+\frac{P_{\mathrm{pgp},\mathrm{app}}^{\mathrm{active}}}{P_{0}\cdot 24\cdot f_{n,\mathrm{cyt}}}\right)\cdot \left(1+\frac{P_{b,\mathrm{app}}^{\mathrm{active}}}{P_{0}\cdot f_{n,b}}\right)+P_{\mathrm{para}}}{P_{\mathrm{trans},A\to B}+P_{\mathrm{para}}}$$

## 3 Materials and methods

### 3.1 The pH-dependence of the ER and determining the transported species

From the expressions for $P_{\mathrm{pgp},\mathrm{app}}^{\mathrm{active}}$ (Eqs. (4), (5)) and Eq. (8) it is evident that if P-gp (or the basolateral transporter) has a preference for a certain species of an ionisable chemical, then efflux would be proportional to the relation of the preferred species fraction (whether $f_{n}$ or $f_{i}$) and $f_{n}$. It has been determined that rather than being well-buffered to remain at pH 7.4 as often suggested, the cytosolic pH changes with external pH (Dahley et al., 2023; Perdikis et al., 1998; Liang et al., 2007). As such, cytosolic pH was estimated as a function of external pH according to the linear relationship determined by Dahley et al. (2023). We postulated that the ER measured as a function of pH can give an indication as to whether increasing or decreasing the available fraction of a specific species has likely affected $P_{\mathrm{pgp}}$, provided that $P_{\mathrm{para}}$ is not dominant. Consequently, for our first aim of investigating a method that could potentially enable the determination of the preferred substrate fraction of a given transporter, we performed MDCK-MDR1 experiments where the ER was determined for the same compound at different external pH values (using the iso-pH method). These experiments were performed across a range of pH values which were determined to not be detrimental to the integrity of the monolayer (Dahley et al., 2024). The results for acidic, basic and non-dissociating compounds were then compared with model fits.

#### 3.1.1 Cell culture

MDCK-II-MDR1 cells were obtained from The Netherlands Cancer Institute (Amsterdam, The Netherlands). The cell medium was Dulbecco's modified Eagle medium (DMEM) (1X) + GlutaMAX™-I supplemented with 10% FBS, 100 U/mL penicillin and 100 μg/mL streptomycin. Cells were maintained at 37 °C in an atmosphere of 5% ${\mathrm{CO}}_{2}$ and passaged twice a week. All chemicals and suppliers can be found in the Supplementary Material.

#### 3.1.2 Selection and preparation of test compounds

Candidate compounds for bidirectional transport studies were selected based on collections of identified P-gp substrates with efflux ratios > 2 as determined by MDCK-MDR1 transport experiments (Troutman and Thakker, 2003; Polli et al., 2001; Éva Hellinger et al., 2012). Three bases were used: 15 μM acebutolol (pKa: 9.18 (Avdeef, 2012)), 100 μM doxorubicin (pKa: 9.56 (Advanced Chemistry Development, 2020)) and 14 μM talinolol (pKa: 9.4). One acid was used: 10 μM etoposide (pKa: 8.53 (Avdeef, 2012)). And finally, two non-dissociating compounds were used: 10 *μ*M digoxin and 50 μM colchicine. Concentrations were chosen to avoid saturation effects and still be within the limits of LC-MS quantification.

#### 3.1.3 Bidirectional transport experiments

Transport experiments were performed as described in Kotze et al. (2024). Briefly, cells (passage 20–40) were seeded onto 12-well PET inserts (CellQART, Northeim, Germany; pore size: 0.4 μm; filter thickness: 11.5 μm, $100\times 10^{6}$ pores/cm^2^) at a density of $1.5\times 10^{5}$ cells/insert. After seeding, cells were maintained as described in Section 3.1.1 and allowed to grow for 4 days. One day before the experiments, the cell medium was refreshed. For $P_{0}$ determination, inserts were pre-incubated with the P-gp inhibitor elacridar (2 μM) for 30 min in HBSS pH 7.4. The transport rates in both directions of the test compounds were determined in duplicate or triplicate for 4–5 different pH values between pH 5 and pH 9. The transport buffer was HBSS buffered with 10 mM MES for pH 5 and 6, 25 mM HEPES for pH 7, 7.4 and 8, and 10 mM TAPS for pH 9. Stock solutions were prepared in the transport buffer and pre-warmed to 37 °C. Additional stock solutions with 2 μM elacridar were prepared at suitable pH values to measure $P_{0}$ values in tandem. The pH of all buffer and stock solutions, as well as the pH of all samples after experiment completion, was controlled with a rapid pH automated pH meter (Hudson Robotics, Inc., Springfield, NJ, USA). Inserts were used in 12-well plates (TPP Techno Plastic Products AG, Trasadingen, Switzerland). The basolateral compartment volume was 1.6 mL, and the apical volume was 0.5 mL. Sampling occurred at 3–4 time intervals. In the A→B direction, at each sampling step the filter was placed in a well with fresh buffer, and the previous basolateral volume was sampled. In the B→A direction, 300 μL was sampled from the apical compartment at each step and replaced with an equal volume of fresh transport buffer (a correction for this volume change was factored into the calculation of $P_{\mathrm{app}}$). Timestep length was determined individually for each compound and direction measured to ensure sink conditions. Plates were kept in an orbital shaking incubator at 450 rpm and 37 °C (Titramax and Inkubator 1000, Heidolph Instruments GmbH & Co. KG, Schwabach, Germany) between sampling steps. The donor compartment was sampled at the final timestep in order to calculate recovery.

#### 3.1.4 Monolayer integrity assessment

The TEER across the monolayer was measured before and after the experiments at 37 °C using an EVOM epithelial tissue volt/ohmmeter (World Precision Instruments Inc., Sarasota, FL, USA). The average TEER was $137\pm 9\;\Omega {\mathrm{cm}}^{2}$ before and $134\pm 19\;\Omega {\mathrm{cm}}^{2}$ after the transport experiments, thereby confirming the integrity of the cell monolayers throughout the experiment. The lucifer yellow (LY)(100 μg/mL) permeability of each insert was also measured to further evaluate monolayer integrity. The fluorescence intensity (Ex: 485 nm, Em: 538 nm) of samples was measured using a SpectraMAX Gemini EM spectrophotometer (Molecular Devices LLC., San Jose, CA, USA). Two out of 130 inserts were excluded from the results due to the LY permeability exceeding the pre-defined threshold of $1.5\times 10^{-6}$ cm/s (Irvine et al., 1999).

#### 3.1.5 Sample analysis and calculations

Samples were analysed with an Infinity II 1260 LC system coupled with a 6420 triple quadrupole 145 with ESI source (Agilent Technologies Inc., Santa Clara, CA, USA). Either a Kinetex® F5 (2.6 μm; 100 Å; 50 ${}^{*}$ 3.0 mm) or A Kinetex® C18 (2.6 μm; 100 Å; 50 ${}^{*}$ 3.0 mm) LC column was used (Phenomenex Inc., Torrance, CA, USA). $P_{\mathrm{app}}$ was calculated from the acceptor compartment concentrations $C_{A}$ measured for at least two consecutive timepoints as follows:

(9) $$P_{\mathrm{app}}\;=\;\frac{C_{A,\mathrm{tx}}-C_{A,\mathrm{tx}-1}}{t_{x}-t_{x-1}}\;\times \;\frac{V_{A}}{A\;\times \;\Delta C}$$

where $\frac{C_{A,\mathrm{tx}}-C_{A,\mathrm{tx}-1}}{t_{x}-t_{x-1}}$ is the change in the cumulative concentration in the acceptor compartment per timestep, $V_{A}$ (cm^3^) is the volume of the acceptor compartment, A (cm^2^) is the filter area, and ΔC (μg/mL) is the concentration difference between the donor and acceptor compartment at each timestep. $P_{\mathrm{app}}$ values for each timestep were corrected with the recovery value for that monolayer as done by Neuhoff (2005). Data are presented as the mean of the recovery-corrected $P_{\mathrm{app}}\;\pm$ standard deviation of 2–4 timestep samples of all replicates. The first timestep in the A→B direction was excluded in order to account for lag time (Heikkinen et al., 2009). The ER was calculated as the ratio of these mean $P_{\mathrm{app}}$ values in the B→A and A→B direction as in Eq. (1) for each pH.

## 4 Results and discussion

### 4.1 The pH-dependence of the efflux ratio

**Fig. 3:**
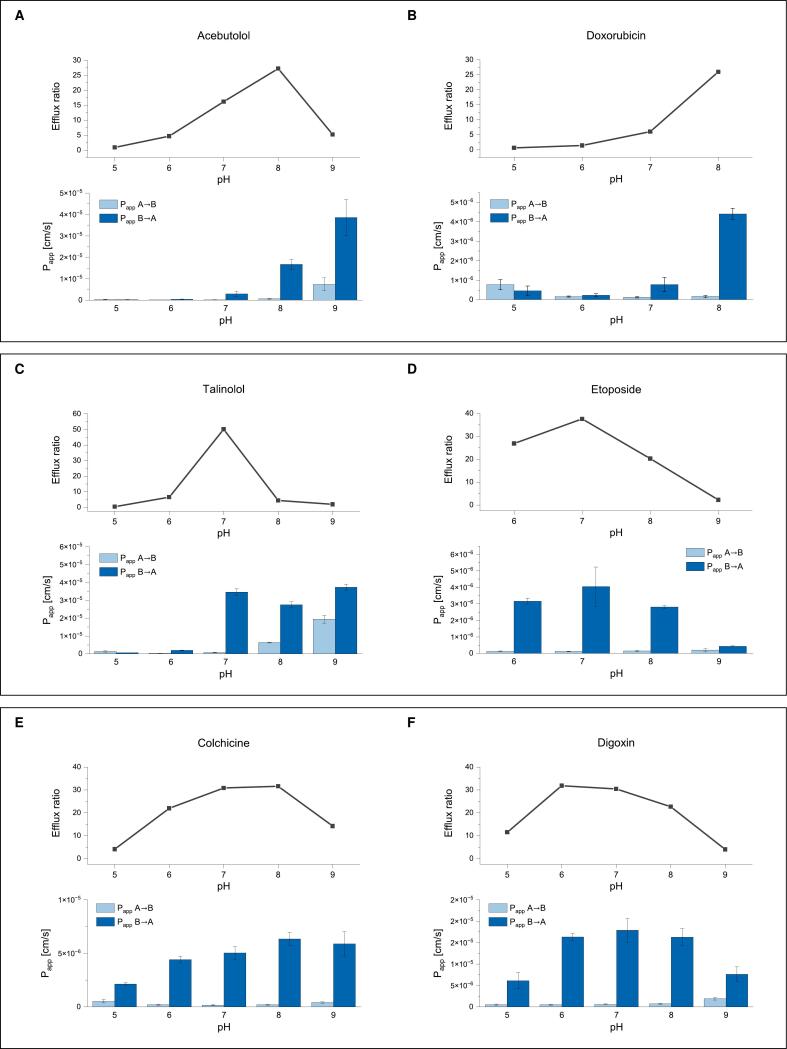
Apparent permeability and efflux ratios as a function of pH for basic, acidic and non-dissociating compounds. Error bars represent standard deviation. The ER (line graph, top panels) is the quotient of mean $P_{\mathrm{app},B\to A}$ over mean $P_{\mathrm{app},A\to B}$ values. A, B and C are basic compounds. D is an acidic compound. E and F are non-dissociating compounds.

Fig. 3 shows the ER values, as well as the $P_{\mathrm{app}}$ values in both directions determined across a range of pH values for each of the six compounds investigated (tabulated values with recovery can be found in the Supplementary Materials). Even though the range of buffer pH values under investigation was determined not to be cytotoxic to the MDCK-II cells within the time range of these assays, it remains unknown whether the transporters themselves could be affected by extreme pH values on opposite ends of the range. As such, any pH effects on the transporters cannot be ruled out definitively.

However, if the transporters are not affected by external pH, it was theorised that non-dissociating compounds would not show great variance in $P_{\mathrm{app},B\to A}$ values, and that the ER would remain relatively stable across the pH range. Fig. 3: E and F depict the results obtained for the two non-dissociating compounds, colchicine and digoxin. The $f_{n}$ for these compounds does not change across the pH range, however, one can see that the $P_{\mathrm{app},B\to A}$ does fluctuate somewhat, and that the ER drops at the extreme ends of the pH range, suggesting that one or both of the transporters may be susceptible to pH effects, a finding that complicates our approach. For the basic compounds acebutolol, doxorubicin and talinolol (Fig. 3: A, B and C), the ER tends to increase with increasing pH (i.e increasing $f_{n}$) in the low pH range. As such, one would expect the opposite trend for acidic compounds, where $f_{n}$ increases with decreasing pH, and this is indeed observed for the anionic compound etoposide (Fig. 3: D).

**Fig. 4:**
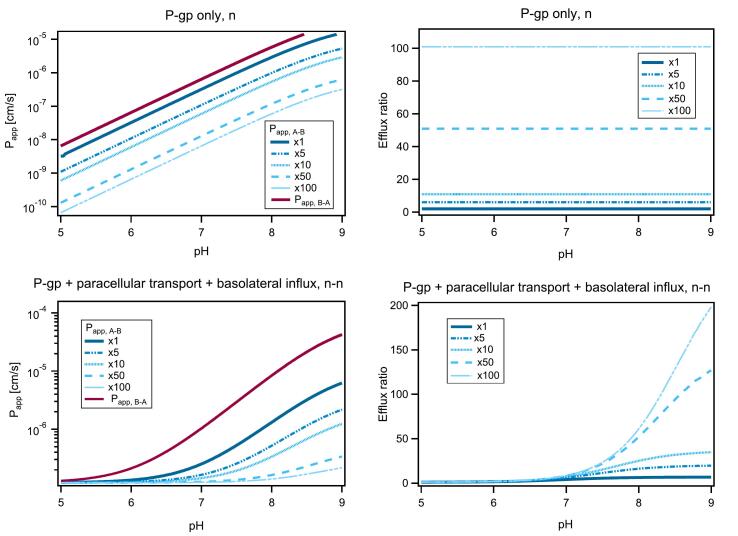
Theoretical curves depicting pH dependence of $P_{\mathrm{app}}$ and ER in the case of P-gp transport only, and in the case of P-gp transport, paracellular transport and basolateral influx for a generic, basic P-gp substrate under the neutral-only assumption. A sensitivity analysis was performed by first setting $P_{\mathrm{pgp}}$ equal to passive diffusion and then intensifying its influence in increments to be 5×, 10×, 50× and 100× passive diffusion. Such curves, always with a step-wise increase in complexity (P-gp only, followed by P-gp + paracellular transport, and lastly P-gp + paracellular transport + basolateral influx) can be found for all three assumptions of transporter preference in the Supplementary Materials, along with further details.

The decrease in ER with decreasing $f_{n}$ is to be expected due to the influence of paracellular transport. In a recent publication, we showed that when paracellular transport dominates the flux in both directions, then the ER reduces to unity (Kotze et al., 2024). Furthermore, even when not dominant, paracellular transport can affect the ER, and the closer it is to dominance, the greater its effect. As such, for the bases (A, B and C) the clear trend of increasing ER with increasing pH (in the low pH range) shows that at lower $f_{n}$ values, paracellular transport competes with transcellular transport, or dominates the flux entirely, which results in lower ER values or an ER of 1, respectively (see Fig. 4 bottom right). Likewise, the acidic etoposide shows the opposite trend, with the same rationale that paracellular transport is more likely to be favoured the smaller the $f_{n}$ available for transcellular transport.

The unexpected drop in ER observed for the bases acebutolol and talinolol at pH 9 and 8, respectively, might indicate at least partially transported ions, since the ER should plateau as soon as paracellular transport is no longer significant under the neutral-neutral assumption (see Fig. 4 bottom right, as well as the Supplementary Materials for graphs depicting ER trends under ion transport assumptions). However, due to the observed decline in active transport even for non-dissociating compounds (Fig. 3 E and F) at high pH values, these trends could also be ascribed to potential effects of these pH values on the transporter. Furthermore, the error in measured $P_{\mathrm{app}}$ is higher at pH 9 for acebutolol, making the interpretation even more uncertain. Evidently, these rough deliberations mean that merely evaluating the $P_{\mathrm{app}}$ and ER data across a pH range for different compounds is an inadequate method of determining the favoured species of P-gp, since it is unavoidable that paracellular transport would affect these data at some pH for any given compound, and that this, combined with the basolateral influx transporter, confounds any conclusions that might otherwise be drawn from such experiments. As a consequence, in Section 4.3 we consider a more holistic view by comparing the $P_{\mathrm{app}}$ values of these compounds with model fits that account for paracellular transport.

### 4.2 Inclusion of the basolateral transporter

In order to determine a reliable $P_{0}$ value for the test compounds, assays using a P-gp inhibitor were performed in parallel with the pH experiments. Contrary to expectations, the $P_{\mathrm{app},B\to A}$ was significantly lower with the inhibitor than without it. This finding is not intuitive because inhibition of P-gp-mediated efflux at the apical membrane should not alter the basolateral membrane's permeability. For transport in the B→A direction, the compound must cross the basolateral membrane first before it becomes available in the cytosol to be transported across the apical membrane by P-gp. Therefore, this passive diffusion across the basolateral membrane would be the rate-limiting step for $P_{\mathrm{app},B\to A}$ even if P-gp accelerates active transport across the across the apical membrane. However, when P-gp facilitated efflux from the apical membrane is inhibited, compounds must cross both membranes via passive diffusion. Thus, in theory, the process should be twice as fast, at most, if it only needs to cross the basolateral membrane when P-gp takes over transport across the second (apical) membrane. This factor of 2 is determined under the unlikely assumption of equal membrane surface areas. The difference would become even smaller when the apical membrane resistance is less than that of the basolateral, due to the likely increased surface area of the apical membrane described in Section 2.1. Yet, empirical data reveal a more substantial increase in $P_{\mathrm{app},B\to A}$ without the inhibitor, routinely exceeding this twofold expectation. An extensive analysis of such data for compounds from our own experiments as well as other literature sources can be found in the Supplementary Material. The significant increase in $P_{\mathrm{app},B\to A}$ without inhibitor implies that transport across the basolateral membrane is also enhanced in some way, thereby increasing the cytosolic concentration available for apical efflux. These findings, along with suggestions from Troutman and Thakker (2003), Li et al. (2012) and others (Agnani et al., 2011; Acharya et al., 2008; Hutter et al., 2014) that a transporter on the basolateral membrane may be responsible for the uptake of compounds into the cytosol of the MDCK cells led to the adaptation of our model for this cell system to include such transport.

### 4.3 Model fits

As described in Section 4.1, pH-dependent evaluations of the ER were not sufficient to make any reliable conclusions as to the efflux transporter's fraction preference due to the influence of paracellular transporter. However, following the refinement of the model to incorporate the basolateral transporter, the subsequent step involved the comparison of model fits generated from our mathematical equations with the experimental data. This was done in another effort to determine the most plausible scenario, taking into account both the model and the empirical data. Global fits of both A→B and B→A transport were performed by employing the varying assumptions described by Eqs. (4), (5) using Igor Pro 7 software (WaveMetrics Inc., Lake Oswego, USA). Recognising that the transporters may have different species preferences, the following three scenarios were evaluated: a) Both transporters act on the neutral fraction, b) both transporters act on the ionic fraction and c) the apical transporter acts on the neutral species, and the basolateral transporter acts on the ionic species. The latter configuration was particularly included in our analysis due to an evaluation of the literature which strongly suggests that the basolateral transporter in epithelial cells could be an ATP-independent organic cation or anion transporter (of the OAT or OCT families) (Hutter et al., 2014; Ito et al., 2005; Galetin et al., 2024; Russel, 2010; Zaïr et al., 2008; Li et al., 2017). Furthermore, some in silico P-gp docking studies assume only transport of unionised compounds by default (Gunaydin et al., 2013; Prajapati et al., 2013), while others that compared both charged and neutral docking reported improved fits when only neutral docking was assumed, regardless of ionisation state (Dolghih et al., 2011; Pajeva et al., 2013; Klepsch et al., 2014). However, there are also studies that found no difference in binding affinities between charged and neutral forms (Kaczor et al., 2020; Cleave et al., 2013). The inconclusive nature of these in silico molecular docking studies highlight the fact that substrate species preference remains an open question.

**Fig. 5:**
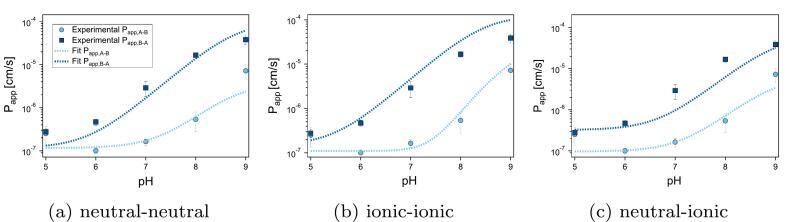
Experimental apparent permeability as a function of pH for acebutolol plotted along with model fits under varying assumptions. A) Both transporters act on the neutral fraction, B) Both transporters act on the ionic fraction and C) The apical transporter acts on the neutral species, and the basolateral transporter acts on the ionic species. Fits were performed using Igor Pro 7 and were weighted by standard deviation.

Fig. 5 depicts the Igor fits (dashed lines) along with the experimental $P_{\mathrm{app}}$ data (markers) in both directions generated for the compound acebutolol. $P_{\mathrm{cyt}}$, $P_{\mathrm{filter}}$, ${\mathrm{pK}}_{a}$, the diffusion coefficient in water $D_{w}$, the surface area of the apical membrane, as well as the individual thickness of the apical $\left(x_{a}\right)$ and basolateral ABL $\left(x_{b}\right)$ were set as fixed parameters. As described in Section 2.1, we used a factor of 24 to account for the increased surface area of the apical membrane, however, due to the uncertainties regarding this value the fits were also performed under the assumption of equal membrane surface areas (i.e S = 1). Though these simulations resulted in nominal changes in the values of the parameters, it did not change the quality of any of the fits. As such, even under this extreme and unlikely assumption, the surface area is not expected to play a significant role for these fits. Furthermore, we recognised that knowing the total ABL thickness alone would not suffice for this study. There is evidence that the ABL thickness on the apical and basolateral sides is not uniform, and that this asymmetry is not predictable (Dahley et al., 2023; Korjamo et al., 2008). Appreciating why these individual thicknesses are critical for the model requires considering that only resistances found downstream from the transporter can be affected by it. Therefore, changes in concentration due to transporter-facilitated efflux would only impact the resistance of the basolateral ABL in the A→B (and thus the apical ABL in the B→A direction). Furthermore, different cell types and experimental systems can result in entirely different values (Dahley et al., 2023). Consequently, we conducted experiments to determine the thickness of the two ABLs separately. Details of these experiments and the resulting ABL sizes can be found in the Supplementary Material.

As is evident from Fig. 5, all the fits generated from the three scenarios describe the data rather well, which lends confidence to the model as a whole. However, none of the assumptions result in a fit that is substantially superior, which makes an unequivocal selection of the most likely case inadvisable. Furthermore, none of the assumptions result in a fit that is unsatisfactory enough to allow for the reasonable exclusion of it as a probable scenario. Although only the fits for acebutolol are depicted in Fig. 5, the results were equally inconclusive for all other compounds evaluated in this study, and our conclusions are based on the critical evaluation of all fits, for all compounds. The corresponding graphs comparing these fits as well as details about the parameters can be found in the Supplementary Material. Though this result is unsatisfying, it is not surprising: the unforeseen inclusion of the basolateral transporter means that there are two free parameters $(P_{\mathrm{pgp}}^{\mathrm{active}}$ and $P_{b}^{\mathrm{active}})$ that must be fitted with rather few data-points, due to the limited viable pH range.

It is worth mentioning that Neuhoff et al. (2003) previously investigated the pH dependence of P-gp substrates using a pH gradient (i.e differing pH values in the basolateral and apical compartments) in Caco-2 cells. It was thought that the recently described concentration shift effects (Dahley et al., 2023) that result from using a pH gradient could complicate analysis and any inferences drawn from such data. To investigate this, we also attempted to fit the Neuhoff et al. (2003) data (for overlapping compounds) with our model, suitably adapted to accommodate the pH gradient. From these fits, the same central conclusion could be drawn: the data cannot be described without the inclusion of a basolateral influx transporter. However, as could be expected for Caco-2 cells, P-gp does not play as significant a role when compared with the fits for the transfected MDCK cells used in our study. These results and a broader analysis can be found in the Supplementary Material. Likewise, Kis et al. (2014) also measured the pH dependence of a single compound, atazanavir, using the gradient-pH method. Due to missing key information regarding ABL sizes and paracellular transport of their set-up, these data could not be fitted. Since atazanavir is neutral throughout the pH range used, changes in $P_{\mathrm{app}}$ were not expected, however Kis et al. reported a pH-dependence of transport. This is likely due to the significant uptake by OATP reported in an earlier study by Kis et al. (2013) for their Caco-2 cells. Breedveld et al. (2007) investigated pH-dependent transport by BCRP in membrane vesicles instead of cells, however found no difference between acids, bases and neutral compounds. Instead, they found increased transport by BCRP at low pH, regardless of substrate dissociation.

### 4.4 Measurement of paracellular transport

In initial fits, we used experimentally obtained values for $P_{\mathrm{para}}$, determined by measuring the $P_{\mathrm{app}}$ of the relevant compound at a pH value where paracellular transport was expected to dominate. Since the compounds in this study are P-gp substrates, elacridar was also used to inhibit P-gp. Under these conditions, the measured $P_{\mathrm{app}}$ is expected to be a measure of $P_{\mathrm{para}}$. However, these assays resulted in $P_{\mathrm{para}}$ values in the range of $1\times 10^{-5}$ to $1\times 10^{-6}$ cm/s, and it was found that the fits could not be performed by fixing the parameter $P_{\mathrm{para}}$ to these experimentally determined values. However, it could fit the data when $P_{\mathrm{para}}$ was set as free parameter, and paracellular transport was invariably estimated to be 0.5 log units lower than the measured value. It was suspected that the use of inhibitor may be affecting the paracellular measurement. We conducted experiments using various other paracellular markers and inhibitors to probe this suspicion, the full details and results of which can be found in the Supplementary Material. Table 1 compares the results obtained for the attempted measurement of $P_{\mathrm{para}}$ for the non-P-gp substrate chlorothiazide without inhibitor, and with three different P-gp inhibitors: elacridar (2 μM), verapamil (100 μM) and cyclosporin A (10 μM).

**Table 1:** Comparison of $P_{\mathrm{app}}$ values and calculated $\log P_{\mathrm{para}}$ for chlorothiazide with various P-gp inhibitors. $P_{\mathrm{app}}$ values are the mean of three timepoints per replicate, for one replicate ± standard deviation.

Compound	$P_{\mathrm{app}}$ $\left[\times 10^{-6}\mathrm{cm}/s\right]$	$\log P_{\mathrm{para}}$	ER
Chlorothiazide A→ B	0.2 ± 0.0	−6.6	1
Chlorothiazide B→A	0.2 ± 0.0	−6.7	
Chlorothiazide A→ B + elacridar	0.9 ± 0.0	−6.0	–
Chlorothiazide A→B + verapamil	1.4 ± 0.1	−5.9	–
Chlorothiazide A→ B + cyclosporin-A	0.7 ± 0.0	−6.1	–

From Table 1 it can be seen that for all three of these common P-gp inhibitors, the measured $\log P_{\mathrm{para}}$ was higher then without any inhibitor. The reason for this is unclear, and it was beyond the scope of this study to systemically investigate this matter further. From these assays and others presented in the Supplementary Materials, we concluded that the method of reducing $f_{n}$ and using inhibitor does not yield reliable measurements for $P_{\mathrm{para}}$. These experiments suggest that using an inhibitor might influence either $P_{\mathrm{para}}$ itself or its measurement. This led to the decision to leave $P_{\mathrm{para}}$ free to be fitted by the model, and it was rather consistently estimated to be within the range of $1\times 10^{-7}$ to $5\times 10^{-7}$ cm/s, which translates to a $\log P_{\mathrm{para}}$ of between −7 and − 6.3. Values which correspond rather more favourably with the $\log P_{\mathrm{para}}$ obtained for non-P-gp substrate compounds measured without inhibitor, the transport of which is expected to be dominated by the paracellular route.

## 5 Conclusions

Data from our experiments provided strong evidence for a basolateral influx transporter which affects the ER and $P_{\mathrm{app}}$ values measured with MDCK-MDR1 cells. This additional transporter was consequently integrated into the full transport model, and the empirical data was fitted with this model under various assumptions of which species the transporters act on. These fits did not result in any one assumption conclusively outperforming the others. However, while clear indications of transporter species preference remain elusive, studying the pH dependence of the ER has provided valuable insights. In this work, we could show the influence of paracellular transport on the ER not only for the extreme case of dominant $P_{\mathrm{para}}$ where the ER reduces to unity, but also for cases where both paracellular transport and transcellular permeation are significant. The slight drop in ER at pH 5 and 9 for non-dissociating compounds suggest that transporters may be affected by more extreme pH values, even if the cells are not. Furthermore, we found that measurement of P-gp substrates with inhibitor and restriction of the $f_{n}$ does not result in accurate measurements of paracellular transport. Ultimately, the combined influences of paracellular transport, the previously unaccounted for basolateral influx transporter, as well as potential pH effects on the transporter impeded analyses of pH-dependence data. Future research could include transporter-specific inhibitors to separate the effects of the apical and basolateral transporters, and to identify the basolateral uptake transporter responsible for affecting MDCK-II ER data. Furthermore, it could also be useful to address the question of transporter species preference through the use of vesicular transport assays instead of cell transport systems (Breedveld et al., 2007). Despite the challenges encountered, our approach represents a significant step in quantifying active transport.

## CRediT authorship contribution statement

**Soné Kotze:** Writing – original draft, Visualization, Investigation, Formal analysis. **Kai-Uwe Goss:** Writing – review & editing, Supervision, Methodology, Conceptualization. **Andrea Ebert:** Writing – review & editing, Methodology, Formal analysis, Conceptualization.

## Declaration of competing interest

The authors declare that they have no known competing financial interests or personal relationships that could have appeared to influence the work reported in this paper.

## Data Availability

Data will be made available on request.

## Appendix A Supplementary data

Supplementary material: Supplementary Materials: I - List of mathematical equations and abbreviations, II - List of chemicals and suppliers, III- Apparent permeability values for pH-dependence assays, IV- Comparison of apparent permeability with and without inhibitor, V- Intrinsic permeability assays, VI- Measurement of ABL thickness, VII- Model fits and parameters of all compounds, VIII-General model curves, IX- Model fits and parameters of pH-gradient data, X- Measurement of paracellular transport Image 1
