# Supplementary material for: The pH-dependence of efflux ratios determined with bidirectional transport assays across cellular monolayers
Source: Int J Pharm X. 2024 Jul 8;8:100269. doi: 10.1016/j.ijpx.2024.100269 (PMC11637191; doi:10.1016/j.ijpx.2024.100269)
Supplement: Supplementary file 1 — Supplementary material: Supplementary Materials: I - List of mathematical equations and abbreviations, II - List of chemicals and suppliers, III- Apparent permeability values for pH-dependence assays, IV- Comparison of apparent permeability with and without inhibitor, V- Intrinsic permeability assays, VI- Measurement of ABL thickness, VII- Model fits and parameters of all compounds, VIII-General model curves, IX- Model fits and parameters of pH-gradient data, X- Measurement of paracellular transport [file mmc1.pdf]

Supplementary Material

The pH dependence of the efflux ratio measured with bidirectional transport assays across cellular monolayers

Soné Kotze, Kai-Uwe Goss, Andrea Ebert

I Mathematical derivations of equations

Table S1: List of mathematical abbreviations

Abbreviation	Description	Reference
C_a	Bulk concentration on the apical side	
$C_{ABL,a}$	ABL concentration on the apical side adjacent to the apical membrane	
$C_{ABL,b}$	ABL concentration on the basolateral side adjacent to the filter	
C_b	Bulk concentration on the basolateral side	
$C_{cyt,a}$	Cytosolic concentration adjacent to the apical membrane	
$C_{cyt,b}$	Cytosolic concentration adjacent to the basolateral membrane	
C_{filter}	Filter concentration adjacent to the basolateral membrane	
x	Thickness of resistance layer	
D_{cyt}	Diffusion coefficient through cytosol: $D_w \cdot 0.05$	Calculation according to Verkman (2002)
D_w	Diffusion coefficient through water: $D_W = 1.348 \cdot 10^{(-4.13-0.453 \cdot \log MW)}$	Calculation according to Avdeef *et al.* (2010)
ER	Efflux ratio: $P_{app,B\rightarrow A}/P_{app,A\rightarrow B}$	
$f_{n,cyt}$	Fraction of neutral species in the cytosol.	Calculation according to Dahley *et al*. (2023)
$f_{n,a}$	Fraction of neutral species on the apical side	Calculated according to Henderson (1913), Hasselbalch (1916) and Escher *et al.* (2020)
$f_{n,b}$	Fraction of neutral species on the basolateral side	See above
$P_{app,A\rightarrow B}$	Experimentally-obtained apparent permeability in the apical to basolateral direction	
$P_{app,B\rightarrow A}$	Experimentally obtained apparent permeability in the basolateral to apical direction	

$P_{ABL,a}$	Permeability of both species through the apical ABL: $P_{ABL,a} = D_w / x_{ABL,a}$	
$P_{ABL,b}$	Permeability of both species through the basolateral ABL: $P_{ABL,b} = D_w / d_{ABL,b}$	
P_{cyt}	Permeability of both species through the cytosol: $P_{cyt} = D_{cyt} / x_{cyt}$	Bittermann and Goss (2017)
P_{filter}	Permeability of both species through the filter	Karlsson *et al*. (1991)
$P_{m,a}$	Permeability of the neutral species through the apical membrane: $PS_{m,a} = P_0 * 24 * f_n$	Palay and Karlin (1959)
$P_{m,b}$	Permeability of the neutral species through the basolateral membrane: $PS_{m,b} = P_0 * f_n$	
P_0	Intrinsic membrane permeability of the neutral species	
P_{para}	Paracellular transport, assumed equal in both directions	
P_{pgp}	Intrinsic permeability of P-gp-mediated efflux	
$P_{pgp,app}^{active}$	Apparent permeability of P-gp-mediated efflux: $P_{pgp} * \mathit{fraction\ of\ species\ Pgp\ acts\ on}$	
P_b^{active}	Intrinsic permeability of influx mediated by basolateral transporter	
$P_{b,app}^{active}$	Apparent permeability of basolateral transporter-mediated influx: $P_b * \mathit{fraction\ of\ species\ basolateral\ transporter\ acts\ on}$	
$P_{trans,A \to B}$	Apparent transcellular permeability, including active transport, diffusion across membranes, and diffusion across the cytosol, in A→B direction	
$P_{trans,B \to A}$	Apparent transcellular permeability, including active transport, diffusion across membranes, and diffusion across the cytosol, in B→A direction	
$S_{ABL,a}^{A \to B}$	Concentration shift effect in apical ABL in basolateral to apical direction	Dahley *et al*. (2023)
$S_{ABL,b}^{B \to A}$	Concentration shift effect in basolateral ABL and Filter in apical to basolateral direction	Dahley *et al*. (2023)
$S_{cyt}^{A \to B}$	Concentration shift factor in cytosol in apical to basolateral direction	Dahley *et al*. (2023)
J	Steady state flux	

For the following derivations it is assumed that the passive permeation of the ionic species through the biological membranes is negligible. Permeation through aqueous films is assumed to be the same for all species, regardless of ionisation state of the compound. The fluxes J (unit: mass/area/time) in the individual compartments of the system described in Figure 2 are described as follows:

$$J_{ABL,a} = P_{ABL,a}\left(C_a - C_{ABL,a}\right) \quad (S1)$$

$$J_{cell} = J_{trans} + J_{para} = P_{trans,A\rightarrow B} * C_{ABL,a} - P_{trans,B\rightarrow A} * C_{filter} + P_{para} * \left(C_{ABL,a} - C_{filter}\right) \quad (S2)$$

$$J_{filter} = P_{filter}\left(C_{filter} - C_{ABL,b}\right) \quad (S3)$$

$$J_{ABL,b} = P_{ABL,b}\left(C_{ABL,b} - C_b\right) \quad (S4)$$

At steady-state, the flux is the same through each compartment:

$$J_{A\rightarrow B} = J_{ABL,a} = J_{cell} = J_{filter} = J_{ABL,b} \quad (S5)$$

The individual fluxes in the compartments comprising the cellular layer are described as follows:

$$J_{m,a} = C_{ABL,a} * f_{n,a} * P_0 * 24 - C_{cyt,a} * \left(f_{n,cyt} * P_0 * 24 + P_{pgp,app}^{active}\right) \quad (S6)$$

$$J_{cyt} = P_{cyt} * \left(C_{cyt,a} - C_{cyt,b}\right) \quad (S7)$$

$$J_{m,b} = P_0\left(f_{n,cyt} * C_{cyt,b} - f_{n,b} * C_{filter}\right) - C_{filter} * P_{b,app}^{active} \quad (S8)$$

At steady-state, the flux is the same through each cellular compartment:

$$J_{trans} = J_{m,a} = J_{cyt} = J_{m,b} \quad (S9)$$

As a consequence of the above flux equations, the permeability through the cell layer in the A ⟶ B direction can be described as:

$$P_{trans,A\rightarrow B} = \frac{1}{\left(1 + \frac{P_{pgp,app}^{active}}{P_0 * 24 * f_{n,cyt}}\right) * \left(\frac{1}{f_{n,a} * P_0} + \frac{1}{\frac{f_{n,a}}{f_{n,cyt}} * P_{cyt}}\right) + \frac{1}{P_0 * 24 * f_{n,a}}}$$
$$= \frac{1}{\left(1 + \frac{P_{pgp,app}^{active}}{P_0 * 24 * f_{n,cyt}}\right) * \left(\frac{1}{f_{n,a} * P_0} + \frac{1}{S_{cyt}^{A\rightarrow B} * P_{cyt}}\right) + \frac{1}{P_0 * 24 * f_{n,a}}} \quad (S10)$$

The permeability through the cell layer in the B ⟶ A direction can be described as follows:

$$P_{trans,B\rightarrow A} = \frac{\left(1 + \frac{P_{pgp,app}^{active}}{f_{n,cyt} * P_0 * 24}\right) * \left(1 + \frac{P_{b,app}^{active}}{f_{n,b} * P_0}\right) * \frac{f_{n,b}}{f_{n,a}}}{\left(1 + \frac{P_{pgp,app}^{active}}{P_0 * 24 * f_{n,cyt}}\right) * \left(\frac{1}{f_{n,a} * P_0} + \frac{1}{\frac{f_{n,a}}{f_{n,cyt}} * P_{cyt}}\right) + \frac{1}{P_0 * 24 * f_{n,a}}}$$

$$= \left(1 + \frac{P_{pgp,app}^{active}}{f_{n,cyt} * P_0 * 24}\right) * \left(1 + \frac{P_{b,app}^{active}}{f_{n,b} * P_0}\right) * \frac{f_{n,b}}{f_{n,a}} * P_{trans,A\rightarrow B} \qquad (S11)$$

Therefore, the full flux equation for transport in the direction A ⟶ B is defined as follows:

$$J_{A\rightarrow B} = \frac{1}{\frac{1}{\frac{(P_{trans,A\rightarrow B} + P_{para})}{(P_{trans,B\rightarrow A} + P_{para})} * P_{ABL,b}} + \frac{1}{\frac{(P_{trans,A\rightarrow B} + P_{para})}{(P_{trans,B\rightarrow A} + P_{para})} * P_{filter}} + \frac{1}{(P_{trans,A\rightarrow B} + P_{para})} + \frac{1}{P_{ABL,a}}} * C_a$$

$$- \frac{\frac{(P_{trans,B\rightarrow A} + P_{para})}{(P_{trans,A\rightarrow B} + P_{para})}}{\frac{1}{\frac{(P_{trans,A\rightarrow B} + P_{para})}{(P_{trans,B\rightarrow A} + P_{para})} * P_{ABL,b}} + \frac{1}{\frac{(P_{trans,A\rightarrow B} + P_{para})}{(P_{trans,B\rightarrow A} + P_{para})} * P_{filter}} + \frac{1}{(P_{trans,a\rightarrow b} + P_{para})} + \frac{1}{P_{ABL,a}}} * C_b \qquad (S12)$$

When expressed in the experimentally obtained metrics, equation S12 is equivalent to:

$$J_{A\rightarrow B} = P_{app,A\rightarrow B} * C_a - P_{app,B\rightarrow A} * C_b \qquad (S13)$$

And at infinite sink conditions (C_b=0), Eq. S12 simplifies to the following:

$$J_{A\rightarrow B} = \frac{1}{\frac{1}{\frac{(P_{trans,A\rightarrow B} + P_{para})}{(P_{trans,B\rightarrow A} + P_{para})} * P_{ABL,b}} + \frac{1}{\frac{(P_{trans,A\rightarrow B} + P_{para})}{(P_{trans,B\rightarrow A} + P_{para})} * P_{filter}} + \frac{1}{(P_{trans,A\rightarrow B} + P_{para})} + \frac{1}{P_{ABL,a}}} * C_a$$

$$= \frac{1}{\frac{1}{S_{ABL,b}^{A\rightarrow B} * P_{ABL,b}} + \frac{1}{S_{ABL,b}^{A\rightarrow B} * P_{filter}} + \frac{1}{(P_{trans,A\rightarrow B} + P_{para})} + \frac{1}{P_{ABL,a}}} * C_a \qquad (S14)$$

Likewise, the full flux equation for transport in the direction B ⟶ A is defined as follows:

$$J_{B\rightarrow A} = -\frac{1}{\frac{1}{\frac{(P_{trans,A\rightarrow B} + P_{para})}{(P_{trans,B\rightarrow A} + P_{para})} * P_{ABL,b}} + \frac{1}{\frac{(P_{trans,A\rightarrow B} + P_{para})}{(P_{trans,B\rightarrow A} + P_{para})} * P_{filter}} + \frac{1}{(P_{trans,A\rightarrow B} + P_{para})} + \frac{1}{P_{ABL,a}}} * C_a$$

$$+ \frac{\frac{(P_{trans,B\rightarrow A} + P_{para})}{(P_{trans,A\rightarrow B} + P_{para})}}{\frac{1}{\frac{(P_{trans,A\rightarrow B} + P_{para})}{(P_{trans,B\rightarrow A} + P_{para})} * P_{ABL,b}} + \frac{1}{\frac{(P_{trans,A\rightarrow B} + P_{para})}{(P_{trans,B\rightarrow A} + P_{para})} * P_{filter}} + \frac{1}{(P_{trans,A\rightarrow B} + P_{para})} + \frac{1}{P_{ABL,a}}} * C_b \qquad (S15)$$

And at infinite sink conditions (C_a=0), Eq. S15 simplifies to the following:

$$J_{B\to A} = \frac{\frac{P_{trans,B\to A}+P_{para}}{P_{trans,A\to B}+P_{para}}}{\frac{1}{\frac{(P_{trans,A\to B}+P_{para})}{(P_{trans,B\to A}+P_{para})}*P_{ABL,b}}+\frac{1}{\frac{(P_{trans,A\to B}+P_{para})}{(P_{trans,B\to A}+P_{para})}*P_{filter}}+\frac{1}{(P_{trans,A\to B}+P_{para})}+\frac{1}{P_{ABL,a}}}*C_b$$

$$= \frac{1}{\frac{1}{P_{ABL,b}}+\frac{1}{P_{filter}}+\frac{1}{(P_{trans,B\to A}+P_{para})}+\frac{1}{P_{ABL,a}*\frac{(P_{trans,B\to A}+P_{para})}{(P_{trans,A\to B}+P_{para})}}}*C_b$$

$$= \frac{1}{\frac{1}{P_{ABL,b}}+\frac{1}{P_{filter}}+\frac{1}{(P_{trans,B\to A}+P_{para})}+\frac{1}{P_{ABL,a}*S_{ABL,a}^{B\to A}}}*C_b \qquad (S16)$$

As per Eq. 1 in the main text, the following relationships are derived for the ER:

$$ER = \frac{(P_{trans,B\to A}+P_{para})}{(P_{trans,A\to B}+P_{para})}$$

$$= \frac{\frac{\left(1+\frac{P_{pgp,app}^{active}}{f_{n,cyt}*P_0*24}\right)*\left(1+\frac{P_{b,app}^{active}}{f_{n,b}*P_0}\right)*\frac{f_{n,b}}{f_{n,a}}}{\left(1+\frac{P_{pgp,app}^{active}}{P_0*24*f_{n,cyt}}\right)*\left(\frac{1}{f_{n,a}*P_0}+\frac{1}{\frac{f_{n,a}}{f_{n,cyt}}*P_{cyt}}\right)+\frac{1}{P_0*24*f_{n,a}}}+P_{para}}{\frac{1}{\left(1+\frac{P_{pgp,app}^{active}}{P_0*24*f_{n,cyt}}\right)*\left(\frac{1}{f_{n,a}*P_0}+\frac{1}{\frac{f_{n,a}}{f_{n,cyt}}*P_{cyt}}\right)+\frac{1}{P_0*24*f_{n,a}}}+P_{para}}$$

$$= \frac{\frac{\left(1+\frac{P_{pgp,app}^{active}}{f_{n,cyt}*P_0*24}\right)*\left(1+\frac{P_{b,app}^{active}}{f_{n,b}*P_0}\right)*\frac{f_{n,b}}{f_{n,a}}}{\left(1+\frac{P_{pgp,app}^{active}}{P_0*24*f_{n,cyt}}\right)*\left(\frac{1}{f_{n,a}*P_0}+\frac{1}{S_{cyt}^{A\to B}*P_{cyt}}\right)+\frac{1}{P_0*24*f_{n,a}}}+P_{para}}{\frac{1}{\left(1+\frac{P_{pgp,app}^{active}}{P_0*24*f_{n,cyt}}\right)*\left(\frac{1}{f_{n,a}*P_0}+\frac{1}{S_{cyt}^{A\to B}*P_{cyt}}\right)+\frac{1}{P_0*24*f_{n,a}}}+P_{para}} \qquad (S17)$$

In the special case where P_{para} is negligible, then equation S17 reduces to:

$$ER = \left(1+\frac{P_{pgp,app}^{active}}{f_{n,cyt}*P_0*24}\right)*\left(1+\frac{P_{b,app}^{active}}{f_{n,b}*P_0}\right)*\frac{f_{n,b}}{f_{n,a}} \qquad (S18)$$

When P_{para} is considered and the the iso-method is applied, then Eq. S17 can be expressed Eq. 9 in the main text. In the special case where P_{para} dominates flux in both the A⟶B and B⟶A direction, then Eq. S17 reduces to unity.

As per Dahley *et al*. (2023), the concentration shift factors used in the above equations are defined as follows:

$$S_{cyt}^{A\rightarrow B} = \frac{f_{n,a}}{f_{n,cyt}} \quad (S19)$$

$$S_{ABL,b}^{A\rightarrow B} = \frac{(P_{trans,A\rightarrow B} + P_{para})}{(P_{trans,B\rightarrow A} + P_{para})} = \frac{1}{S_{ABL,a}^{B\rightarrow A}} \quad (S20)$$

$$S_{ABL,a}^{B\rightarrow A} = \frac{(P_{trans,B\rightarrow A} + P_{para})}{(P_{trans,A\rightarrow B} + P_{para})} \quad (S21)$$

Calculation of neutral fraction (f_n)

Monovalent base:

$$f_n = \frac{1}{10^{pK_{a1}-pH} + 1} \quad (S22)$$

Divalent base:

$$f_n = \frac{1}{10^{pK_{a1}+pK_{a2}-2\cdot pH} + 10^{pK_{a1}-pH} + 1} \quad (S23)$$

Monovalent acid:

$$f_n = \frac{1}{10^{-pK_{a1}+pH} + 1} \quad (S24)$$

For more complex speciation, see Escher *et al.* (2020).

II Chemicals

Table S2: List of chemicals and suppliers.

Chemical	Supplier
**Cell Culture**	
Dulbecco's modified Eagle medium (DMEM); high glucose, GlutaMAX™ Supplement	Life Technologies Ltd., Paisley, UK
Penicillin Streptomycin ([+] 10,000 Units/mL Penicillin, [+] 10,000 µg/mL Streptomycin)	Life Technologies Corporation, Grand Island, NY, USA
Fetal Bovine Serum (FBS)	Life Technologies Corporation, Grand Island, NY, USA
**Monolayer Efflux Studies**	
Bidirectional pH-dependence assays	
Hanks' balanced salts solution (HBSS);<br>w/ Calcium, Magnesium, Sodium Bicarbonate	Biowest SAS, Nuaillé, France
4-Morpholineethanesulfonic acid (MES)	Sigma-Aldrich, Co., St. Louis, MO, USA
N-(2-Hydroxyethyl)piperazine-N'-(2-ethanesulfonic acid) (HEPES)	Sigma-Aldrich, Co., St. Louis, MO, USA
N-[Tris(hydroxymethyl)methyl]-3-aminopropansulfonsäure, [(2-Hydroxy-1,1-bis(hydroxymethyl)ethyl)amino]-1-propansulfonsäure (TAPS)	Sigma-Aldrich, Co., St. Louis, MO, USA
Lucifer Yellow CH dilithium salt	Sigma-Aldrich, Co., St. Louis, MO, USA
Acebutolol hydrochloride	Sigma-Aldrich, Co., St. Louis, MO, USA
(±)-Talinolol	Cayman Chemical, Ann Arbor, MI, USA
Doxorubicin hydrochloride	Thermo Fisher Scientific, Waltham, MA, USA
Digoxin	Sigma-Aldrich, Co., St. Louis, MO, USA
Colchicine	Thermo Fisher Scientific, Waltham, MA, USA
Etoposide	Sigma-Aldrich, Co., St. Louis, MO, USA
P_{para} and P_0 assays	
Elacridar (GF120918)	Sigma-Aldrich, Co., St. Louis, MO, USA
Cyclosporin A	Sigma-Aldrich, Co., St. Louis, MO, USA
Verapamil hydrochloride	Sigma-Aldrich, Co., St. Louis, MO, USA
DMSO	Th. Geyer GmbH & Co. KG, Renningen, Germany
Chlorothiazide	Sigma-Aldrich, Co., St. Louis, MO, USA
Atenolol	Sigma-Aldrich, Co., St. Louis, MO, USA
Furosemide	Sigma-Aldrich, Co., St. Louis, MO, USA
**Measurement of ABL thickness**	
Indomethacin	Sigma-Aldrich, Co., St. Louis, MO, USA

III P_{app} values for pH-dependence bidirectional assays

Table S3: Recovery-corrected P_{app} values, ER and recovery for all compounds at various pH values using the iso-method in MDCK-MDR1 cells.

Compound	pH	$P_{app, A\rightarrow B}$ [x 10^{-6} cm/s]	$logP_{app, A\rightarrow B}$ [P in cm/s]	$P_{app, B\rightarrow A}$ [x 10^{-6} cm/s]	$logP_{app, B\rightarrow A}$ [P in cm/s]	ER	Recovery [%]
**Acebutolol**	5	0.31 ± 0.11	-6.51	0.28 ± 0.05	-6.56	0.9	90-106
	6	0.10 ± 0.02	-7.00	0.47 ± 0.07	-6.33	4.7	88-120
	7	0.18 ± 0.02	-6.74	2.92 ± 1.12	-5.54	16.2	88-102
	8	0.61 ± 0.28	-6.21	16.68 ± 2.4	-4.78	27.2	78-83
	9	7.41 ± 3.04	-5.13	38.55 ± 8.5	-4.41	5.2	72-79
**Colchicine**	5	0.52 ± 0.15	-6.29	2.12 ± 0.14	-5.67	4.1	100-120
	6	0.20 ± 0.04	-6.70	4.42 ± 0.33	-5.35	21.9	78-109
	7	0.16 ± 0.04	-6.79	5.03 ± 0.58	-5.30	30.8	98-110
	8	0.20 ± 0.05	-6.70	6.33 ± 0.62	-5.20	31.6	96-102
	9	0.41 ± 0.06	-6.38	5.89 ± 1.14	-5.23	14.2	92-100
**Digoxin**	5	0.53 ± 0.15	-6.27	6.12 ± 1.90	-5.21	11.5	71-116
	6	0.51 ± 0.12	-6.29	16.33 ± 0.8	-4.79	31.8	74-88
	7.4	0.59 ± 0.11	-6.23	17.83 ± 2.8	-4.75	30.4	73-83
	8	0.72 ± 0.13	-6.14	16.26 ± 2.0	-4.79	22.7	73-76
	9	1.90 ± 0.32	-5.72	7.63 ± 1.73	-5.12	4.0	75-82
**Doxorubicin**	5	0.77 ± 0.26	-6.34	0.46 ± 0.25	-6.34	0.6	94-100
	6	0.16 ± 0.03	-6.79	0.23 ± 0.08	-6.64	1.4	90-96
	7	0.13 ± 0.03	-6.89	0.78 ± 0.36	-6.11	6.0	88-93
	8	0.17 ± 0.05	-6.77	4.40 ± 0.29	-5.36	26.0	64-71
**Etoposide**	6	0.12 ± 0.02	-6.93	3.16 ± 0.19	-5.50	26.8	92-97
	7.4	0.11 ± 0.02	-6.97	4.05 ± 1.18	-5.39	37.6	98-108
	8	0.14 ± 0.04	-6.86	2.80 ± 0.08	-5.55	20.2	83-98
	9	0.19 ± 0.08	-6.71	0.43 ± 0.03	-6.37	2.2	60-71
**Talinolol**	5	1.33 ± 0.36	-5.88	0.57 ± 0.02	-6.25	0.4	94-101
	6	0.30 ± 0.04	-6.53	1.92 ± 0.19	-5.72	6.5	98-102
	7.4	0.69 ± 0.14	-6.16	34.48 ± 1.9	-4.46	50.1	97-100
	8	6.23 ± 0.22	-5.21	27.41 ± 1.6	-4.56	4.4	94-99
	9	19.31 ± 2.2	-4.71	37.18 ± 1.8	-4.43	1.9	91-110

IV Comparison of $P_{app, B\text{-}A}$ with and without inhibitor

In Section 4.2 of the main text, we outlined the experimental evidence suggesting that a basolateral influx transporter is involved in transport across the monolayer. This conclusion was primarily drawn from a comparison between $P_{app,B\text{-}A}$ values with P_{app} derived from experiments where P-gp was inhibited. From our own experiments we observed that $P_{app,B\text{-}A}$ was consistently greater than P_{app} with inhibitor by more than a factor of 2. However, since we only had data for a limited number of

compounds, we wanted to establish whether this was a typical trend before drawing any conclusions. As such, we evaluated data from 12 other literature sources that published such values for compounds with an ER greater than 2. Table S4 shows this collection of data, where the two P_{app} values are compared and the magnitude of the difference calculated. These P_{app} values were all determined with the iso-pH method and always at same pH with and without inhibitor. On the basis of these data and the factor differences that were calculated for all compounds, the suspicion that a basolateral influx transporter may be involved was affirmed. The model was therefore adapted to include such transport facilitated by an influx pump situated in the basolateral membrane, and it was found that this inclusion also improved the model fits shown in the following section. For these data, experiments were always performed at pH 7.4 using the iso-method, with the exception of some compounds from our own experiments which have been performed at different pH values (still using the iso-method) to avoid ABL-limitation. These pH differences explain varying P_{app} values for the same compound. The exceptions in pH are indicated in the table footnote.

Table S4: $P_{app,\,B\text{-}A}$ and P_{app} values with inhibitor from various sources for compounds with an ER > 2 in MDCK-MDR1 cells.

Compound	$P_{app,\,B\rightarrow A}$ [x 10^{-6} cm/s]	$P_{app,\,inhibitor}$ [x 10^{-6} cm/s]	Factor difference	Source
5-Hydroxymethyl Tolterodine	63	11	6	Callegari et al. (2011)
Acebutolol[a]	16.7	5.03	3	This study
Acebutolol[a]	16.7	3.16	5	This study
Acebutolol	17.2	3.73	5	Troutman and Thakker (2003)
Amprenavir	98.7	40.9	2	Polli *et al.* (2001)
Amprenavir	83.4	40.1	2	Doan *et al.* (2002)
Brompheniramine	118.8	76.8	2	Obradovic *et al.* (2007)
Cetirizine	21.7	4	5	Doan *et al.* (2002)
Cetirizine	18.3	2.46	7	Obradovic *et al.* (2007)
Chlorpheniramine	93.7	66.2	1	Obradovic *et al.* (2007)
Cimetidine	1.89	0.515	4	Doan *et al.* (2002)
Clemastine	156.4	49.5	3	Obradovic *et al.* (2007)
Colchicine[a]	6.33	1.73	4	This study
Colchicine[a]	6.33	0.94	7	This study
Colchicine[a]	4.62	0.53	9	This study
Colchicine	11.9	1.64	7	Troutman and Thakker (2003)
Digoxin	17.0	2.53	7	This study
Digoxin	35.7	7.5	5	Troutman and Thakker (2003)
Digoxin	15.2	2.04	7	Chang *et al.* (2006)
Digoxin	9.75	1	10	Wang *et al.* (2005)
Digoxin	8.15	1.39	6	Taub *et al.* (2005)
Diphenhydramine	103.7	77.8	1	Doan *et al.* (2002)
Dipyridamole	43.3	16.3	3	Wang *et al.* (2005)
Eletriptan	66.3	42.9	2	Doan *et al.* (2002)
Etoposide	10.4	0.66	16	Troutman and Thakker (2003)

Etoposide	1.8	0.3	6	Wang *et al.* (2005)
Fexofenadine	1.34	0.27	5	Obradovic *et al.* (2007)
Indinavir	26.9	8.5	3	Doan *et al.* (2002)
Labetalol	18.19	1	18	Wang *et al.* (2005)
Labetalol	36.2	7.37	5	Doan *et al.* (2002)
Lamiduvine	0.18	0.11	2	De Souza *et al.* (2009)
Loperamide	42.64	12	4	Wang *et al.* (2005)
Loperamide	77.3	45.6	2	Doan *et al.* (2002)
Loratadine	35	39.64	1	Obradovic *et al.* (2007)
Methylprednisolone	94.3	28.48	3	Troutman and Thakker (2003)
Methysergide	75.3	39.4	2	Doan *et al.* (2002)
Morphine	14.4	8.62	2	Troutman and Thakker (2003)
Nelfinavir	78.6	19.7	4	Doan *et al.* (2002)
Neostigmine	1.54	0.852	2	Doan *et al.* (2002)
Prednisolone	84.9	27.22	3	Troutman and Thakker (2003)
Pyrilamine	100.4	73.8	1	Obradovic *et al.* (2007)
Quinidine	156	25.6	6	Troutman and Thakker (2003)
Quinidine	66.88	0.176	380	Gertz *et al.* (2010)
Quinidine	114.82	10	12	Wang *et al.* (2005)
Ranitidine	3.29	1.25	3	Troutman and Thakker (2003)
Rhodamine 123	15.3	1.16	13	Troutman and Thakker (2003)
Rhodamine 123	10.17	1.173	9	Tang *et al.* (2004)
Rhodamine 123	7.63	2.2	3	Wang *et al.* (2005)
Ritonavir	23.6	3.91	6	Troutman and Thakker (2003)
Saquinavir	108	12.6	9	Troutman and Thakker (2003)
Saquinavir	39.5	22	2	Doan *et al.* (2002)
Sotolol	1.05	0.2	5	Liu *et al.* (2012)
Talinolol[a]	27.4	6.10	5	This study
Talinolol[a]	27.4	4.73	6	This study
Talinolol	22.8	2.69	9	Troutman and Thakker (2003)
Taxol/Paclitaxel	62.9	7.33	9	Troutman and Thakker (2003)
Taxol/Paclitaxel	21.02	1.65	13	Taub *et al.* (2005)
Terfenadine	42.1	28.5	1	Doan *et al.* (2002)
Terfenadine	11.43	2.39	5	Obradovic *et al.* (2007)
Verapamil	93.2	38.2	2	Troutman and Thakker (2003)
Vinblastine	35.7	17.7	2	Troutman and Thakker (2003)
Vinblastine	13.22	1.92	6	Taub *et al.* (2005)
Vinblastine	10.77	1.2	9	Wang *et al.* (2005)
Ximelagatran	39	7	6	Eriksson *et al.* (2006)
Zidovudine	9.98	3.26	3	De Souza *et al.* (2009)

[a] Performed at varying pH with the iso-method

V P_0 assays

Membrane permeability, P_m, can be determined from P_{app}, provided that P_{app} is not ABL-limited or dominated by paracellular transport. Where possible, assays were performed in parallel with the pH dependence experiments to determine the P_0 of the measured compounds. The P_0 assays were carried out similarly to the bidirectional transport experiments described in Section 3 of the main text, however with minor changes. Firstly, chemical stock solutions also contained the P-gp inhibitor elacridar (2 µM) to ensure that measured transport rates would not be affected by active efflux. Because of its poor aqueous solubility, elacridar was first dissolved in DMSO. However, the final DMSO concentration of the stock solution did not exceed 0.01 %. Any potential gradient effects between the two compartments was mitigated by adding the same concentration of DMSO to the transport buffer. Secondly, the pH values of these assays were chosen to avoid ABL limitation or paracellular dominance. Details and the results of these assays can be found in Table S5. P_{app} represents the recovery-corrected mean values for at least two timesteps per replicate ± standard deviation, with one replicate performed in each direction. P_0 was extracted from $P_{app,\,A\longrightarrow B}$ (unless stated otherwise). Though not expected to have significant effects at this pH, the cytosol and ABL was considered in the calculation of P_0. However, P_{para} was not explicitly accounted for due to the choice of pH.

Table S5: P_{app} with inhibitor for both directions, ER and calculated $logP_o$ for measured compounds. P_{app} represent the mean of 2-3 timepoints of one replicate ± standard deviation.

Compound	pH	$P_{app,\,A\longrightarrow B}$ [x 10^{-6} cm/s]	$P_{app,\,B\longrightarrow A}$ [x 10^{-6} cm/s]	ER	Recovery [%]	pK_a	f_n [%]	$LogP_0$ [a] [P in cm/s]
**Talinolol** (14 µM) + **Elacridar** (2 µM)	8	6.6 ± 0.3	4.7 ± 0.5	0.8	80	9.4	5	-3.8
**Etoposide** (8.5 µM) + **Elacridar** (2 µM)	7.4	0.41 ± 0.1	0.33 ± 0.1	0.8	90-100	*8.53*[b,c]	84	-6.3
**Digoxin** (10 µM) + **Elacridar** (2 µM)	7.4	2.5 ± 0.2	n/A	n/A	74	-	100	-5.6
**Colchicine** (25 µM) + **Elacridar** (2 µM)	8	1.2 ± 0.4	1.7 ± 0.9	1.5	94-97	-	100	-5.9
**Acebutolol** (15 µM) + **Elacridar** (2 µM)	8	5.7 ± 0.6	5.0 ± 0.5	0.9	91-97	9.18[c]	6	-4.0

[a] Calculated from $P_{app,\,A\longrightarrow B}$.

[b] Acidic pK_a. All non-italic values are basic.

[c] Determined 25 ^{0}C, from Avdeef (2012)

VI Measurement of individual ABL thickness

Transport assays in the A⟶B direction were performed as described in the methods section of the main text. Indomethacin (1 µg/mL, 0.01% DMSO) was used as a non-substrate chemical with relatively high hydrophobicity ($logK_{hex/water}$: 0.58), and pH values of the transport buffer were chosen in order to ensure ABL limitation. Samples were taken every 10 min for 40 min. The total ABL thickness was determined from the upper plateau obtained for testosterone using the iso-pH method in Kotze *et al.* (2024). For the CellQart filters and under the shaking conditions (450 rpm) used in our consistent set-up, the total ABL thickness was determined to be 426 µM. The individual apical and basolateral ABL

thickness was consequently determined by fitting the indomethacin data where a pH-gradient method was used. This method exploits the fact that the permeation across the basolateral ABL is increased by a concentration shift factor $S_{ABL,b}^{A\longrightarrow B}$ which amounts to $f_{n,a}/f_{n,b}$, provided that there is no active transport and a pH difference between the apical and basolateral ABL. At pH 5⟶ 7.4, this means that the permeation across the basolateral ABL is increased by a factor of 221. Thus, in comparison to the apical ABL, it no longer poses a significant resistance and the apical ABL becomes the dominant resistance. Thus, it is possible to extract both individual ABL sizes experimentally. Two replicates were performed under two conditions: pH 5 ⟶ 7.4 and pH 5.5 ⟶ 7.4. The basolateral pH was 7.4 in both cases, and the apical pH was 5 and 5.5, respectively. Using the Igor Pro 7 software, the apical ABL was fitted to be 133 µM, and consequently the basolateral ABL was determined to be 293 µM.

VII Model fits and parameters of all compounds

As described in Section 4.3 of the main text, global fits in both transport directions were performed under the three varying assumptions described by Equations 4-5 using the Igor Pro 7 software. Figures S1-S4 depict the Igor fits (dashed lines) along with the experimental P_{app} data (markers) in both directions generated for the remaining compounds not presented in the main text. Each figure is accompanied by a parameter table (Tables S6-S11) that depicts the values of both the constrained and unconstrained parameters used in the fits. Figures S1 and S2 show the results for the other two basic compounds, talinolol and doxorubicin. As with acebutolol in the main text, a relatively good fit was obtained under all three assumptions for doxorubicin. For talinolol, none of the scenarios seem to fit the data especially well. When the fits for talinolol are generated by masking the pH 9 data, a better fit is achieved for the neutral-neutral scenario only, showing a much improved fitted curve for $P_{app,B\text{-}A}$. Tables S6-S8 show the parameters obtained from all fits for the three basic compounds. The bottom rows show the constrained parameters, while the first three rows in bold are the values obtained for the parameters that were left free to be fitted by the model, unless otherwise indicated. It is clear that for all three basic compounds, changing the assumption of which species the transporters act on results in slight changes in the values of the estimated parameters, however the overall quality of the fit between the three assumptions remains relatively similar. As stated in the main text, comparison of these fits performed for the basic compounds does not produce an obvious prime candidate, nor a fit poor enough to conclusively rule out any of the assumptions.

For the non-dissociating compounds (in absence of significant ionic fraction that could be transported), only the neutral-neutral scenario was fitted. For both non-dissociating compounds digoxin (Figure S4 B and Table S10), and colchicine (Figure S4 A and Table S11), the data suggests a slight decline of ER at extreme pH values of 5 and 9. This can only be explained by less effective transport at extreme pH values, since the neutral fraction of the compounds does not change. These pH-related transporter effects can thus not be reflected by the fit. However, complications arise for the acidic compound etoposide. Under the neutral-neutral assumption (both transporters preferentially transport the neutral species), the model produced reasonable fits. For etoposide, however, the fits from the remaining assumptions (all involving the ionic fraction to various degrees) either results in unreasonably high P_{pgp} estimates or abnormally low P_{para} values (See Figure S3 and Table S9). In this case, low estimates of P_{para} simply imply that paracellular transport is not expected to play a significant role. This unrealistic result could be tempered by placing constraints on the free parameters, which only allowed estimates within the reasonable range. High P_{pgp} estimates could stem from the fit attempting to artificially boost P-gp activity to compensate for the very low basolateral transport,

which is determined by the low $P_{app,\ B\longrightarrow A}$ at pH 9. This compensation is not efficient, since the basolateral resistance in the B⟶A direction is not affected by the transport through the apical membrane. As such, the scenarios involving active transport of the ionic species show a worse fit than the neutral scenario. For etoposide, the neutral-neutral scenario seems to fit the data best, mainly because it can explain the steep drop of the $P_{app,\ B\longrightarrow A}$ at pH 9. However, if the pH 9 datapoint is not included in the evaluation (as we cannot rule out less effective active transport due to extreme pH), the fit performs equally well for scenarios where ionic transport is involved.

The complications that arise under the ionic-ionic and neutral-ionic assumptions for certain compounds could thus be a result of these assumptions being false, and that the model could not fit the experimental data under their limit. However, it could also be as a result of the effect that the rather more extreme pH values of 5 and 9 may have on the transporters, as observed for the non-dissociating compounds. Furthermore, we acknowledge the difficulty of attempting to fit three parameters based on the few datapoints that can be experimentally obtained within the limits of the usable pH range. With these uncertainties, we restate the stance taken in the main text that selecting or discarding any of the assumptions on the basis of these fits would not be prudent, since it has become clear in our studies that the situation is rather more complex than first anticipated.

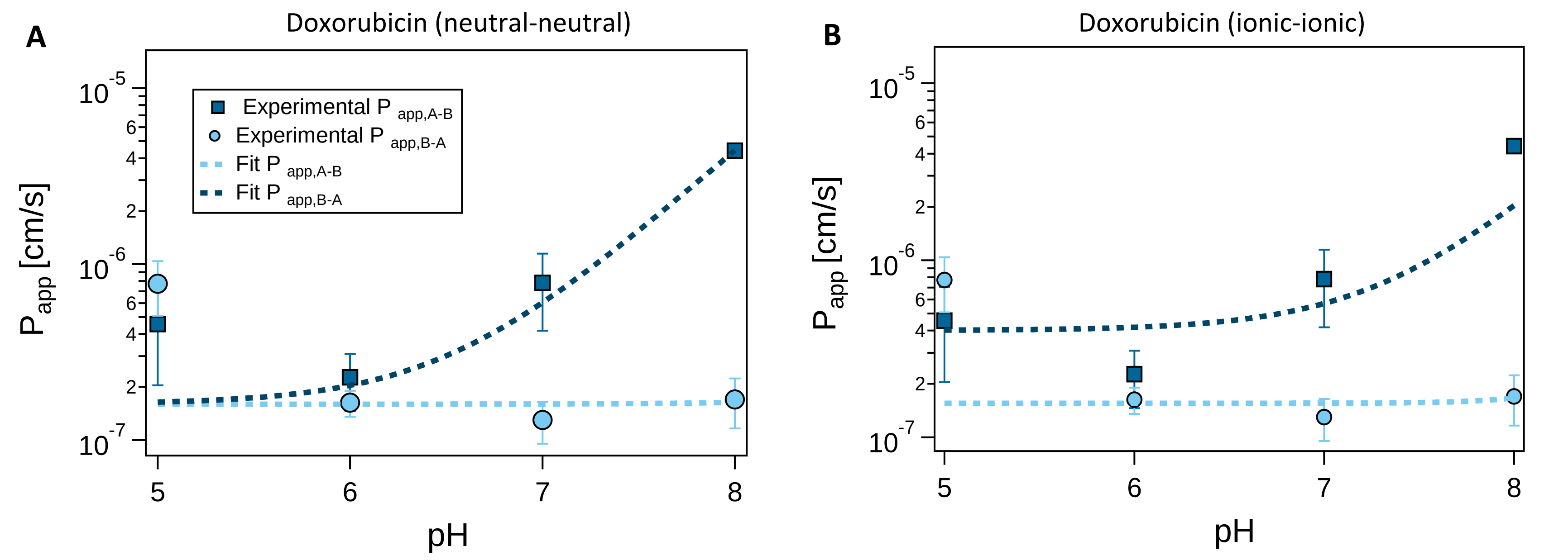

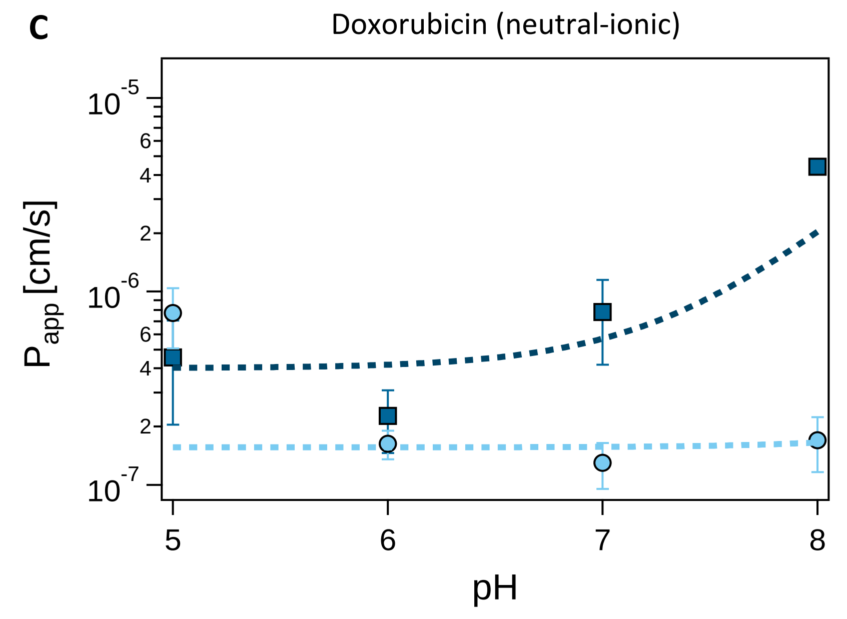

Figure S1: Experimental apparent permeability as a function of pH for doxorubicin plotted along with model fits under varying assumptions. Experimental $P_{app,A \to B}$ (light blue markers) and $P_{app,B \to A}$ (dark blue markers) represent the mean of at least two timepoints and two replicates (n=2), corrected for recovery. Error bars represent standard deviation. The fits were performed using Igor Pro 7 and are shown as dashed lines. All recovery-corrected P_{app} values were included in the fit and weighted by standard deviation. Fits were performed under three different assumptions: A) Both transporters act on the neutral fraction, B) Both transporters act on the ionic fraction and C) The apical transporter acts on the neutral species, and the basolateral transporter acts on the ionic species.

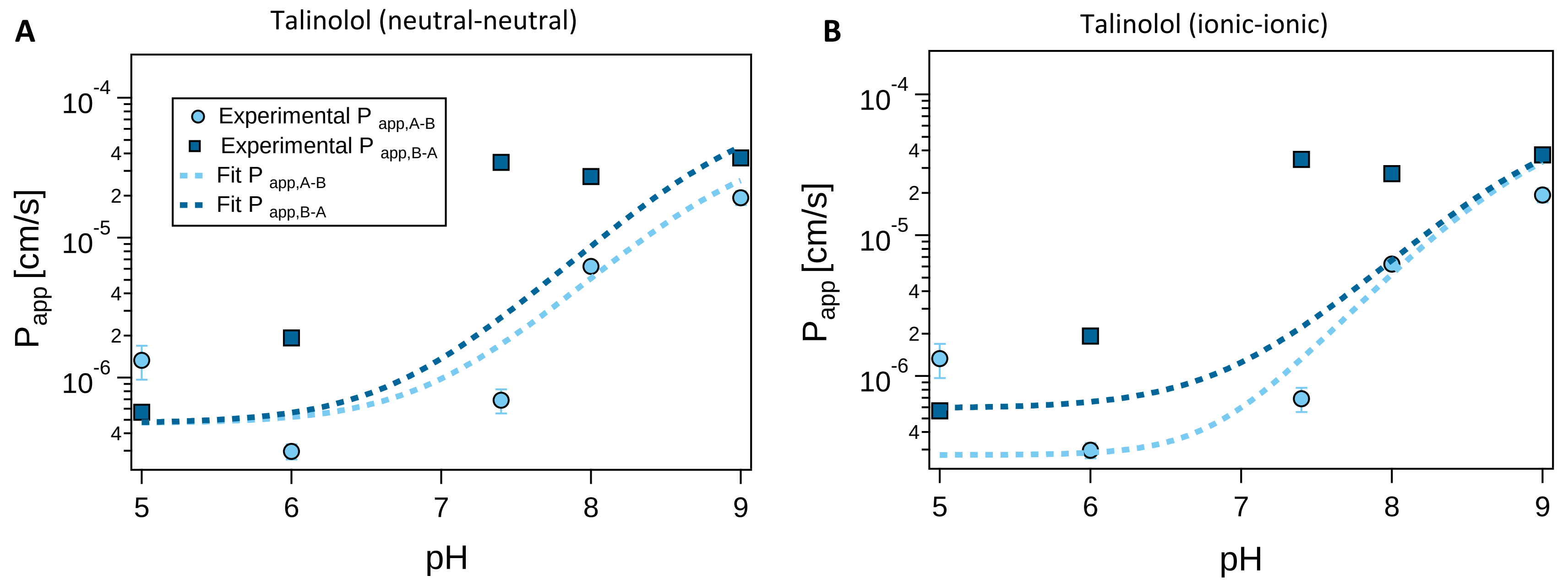

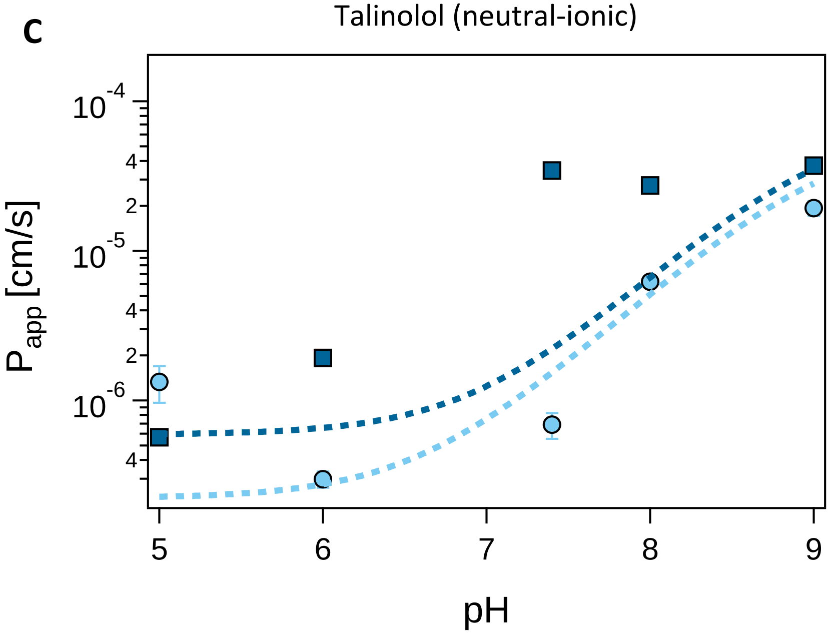

Figure S2: Experimental apparent permeability as a function of pH for talinolol plotted along with model fits under varying assumptions. Experimental $P_{app,A\rightarrow B}$ (light blue markers) and $P_{app,B\rightarrow A}$ (dark blue markers) represent the mean of at least two timepoints and two replicates (n=2), corrected for recovery. Error bars represent standard deviation. The fits were performed using Igor Pro 7 and are shown as dashed lines. All recovery-corrected P_{app} values were included in the fit and weighted by standard deviation. Fits were performed under three different assumptions: A) Both transporters act on the neutral fraction, B) Both transporters act on the ionic fraction and C) The apical transporter acts on the neutral species, and the basolateral transporter acts on the ionic species.

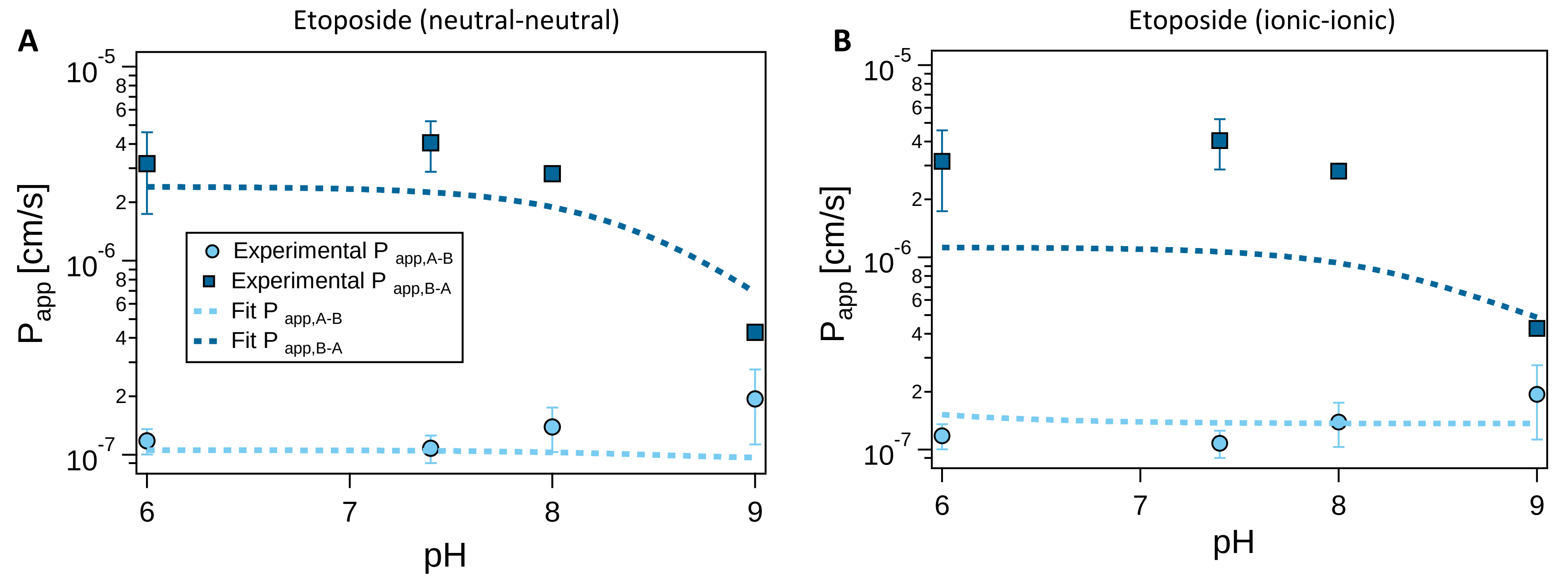

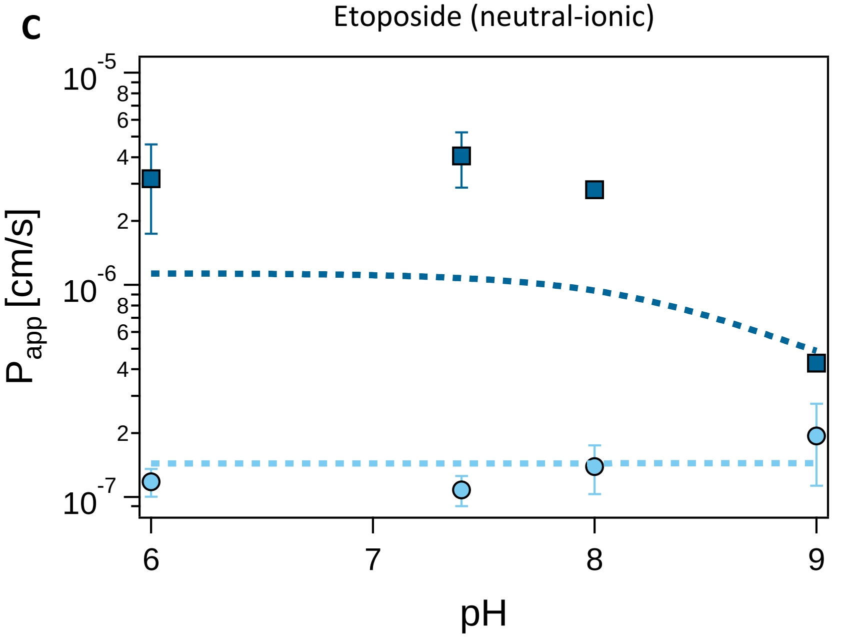

Figure S3: Experimental apparent permeability as a function of pH for etoposide plotted along with model fits under varying assumptions. Experimental $P_{app,A\rightarrow B}$ (light blue markers) and $P_{app,B\rightarrow A}$ (dark blue markers) represent the mean of at least three timepoints and two replicates (n=2), corrected for recovery. Error bars represent standard deviation. The fits were performed using Igor Pro 7 and are shown as dashed lines. All recovery-corrected P_{app} values were included in the fit and weighted by standard deviation. Fits were performed under three different assumptions: A) Both transporters act on the neutral fraction, B) Both transporters act on the ionic fraction and C) The apical transporter acts on the neutral species, and the basolateral transporter acts on the ionic species.

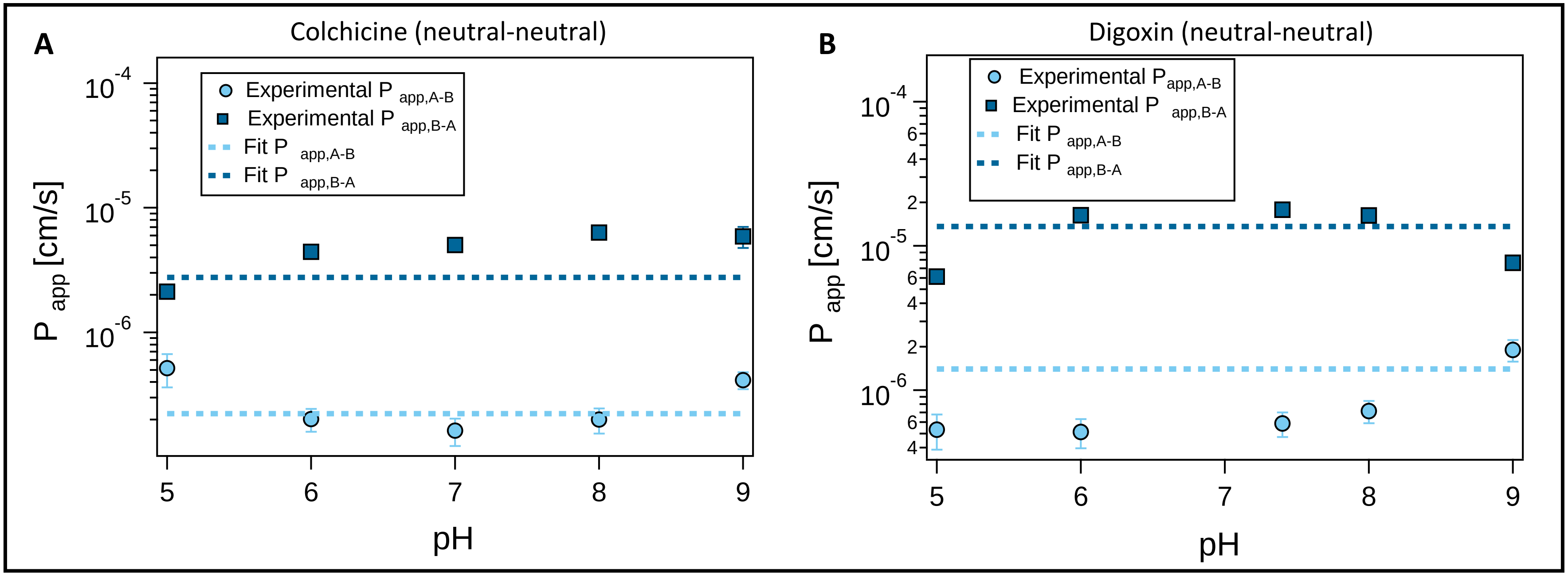

Figure S4: Experimental apparent permeability as a function of pH for A: colchicine and B: digoxin plotted along with model fits. Experimental $P_{app,A\rightarrow B}$ (light blue markers) and $P_{app,B\rightarrow A}$ (dark blue markers) represent the mean of at least two timepoints and two replicates (n=2), corrected for recovery. Error bars represent standard deviation. The fits were performed using Igor Pro 7 and are shown as dashed lines. All recovery-corrected P_{app} values were included in the fit and weighted by standard deviation. Fits were performed under the assumption that both transporters act on the neutral fraction.

Table S6: Parameter table for **Acebutolol.**

Fit Parameters	**A:** Neutral-Neutral	**B:** Ionic-Ionic	**C:** Neutral-Ionic
$P_{pgp,app}^{active}$ (cm/s)	**2.40E-02**	**8.39E-02**	**2.04E-02**
$P_{b,app}^{active}$ (cm/s)	**1.43E-04**	**3.42E-08**	**2.27E-07**
P_{para} (cm/s)	**1.16E-07**	**1.11E-07**	**9.62E-08**
P_{filter} (cm/s)	7.83E-04	7.83E-04	7.83E-04
P_{cyt} (cm/s)	1.19E-03	1.19E-03	1.19E-03
P_0 (cm/s)	9.97e-05	9.97e-05	9.97e-05
x_a (cm)	0.0133	0.0133	0.0133
x_b (cm)	0.0293	0.0293	0.0293
D_w (cm^2/s)	7.16e-06	7.16E-06	7.16E-06
pK_a	9.18	9.18	9.18
S	24	24	24

Table S7: Parameter table for **Talinolol.**

Fit Parameters	**A:** Neutral-Neutral	**B:** Ionic-Ionic	**C:** Neutral-Ionic
$P_{pgp,app}^{active}$ (cm/s)	**1.20E-03**	**4.54E-05**	**1.01E-03**
$P_{b,app}^{active}$ (cm/s)	**6.37E-05**	**3.16E-07**	**3.82E-07**
P_{para} (cm/s)	**4.73E-07**	**2.76E-07**	**2.22E-07**
P_{filter} (cm/s)	7.56E-04	7.56E-04	7.56E-04
P_{cyt} (cm/s)	1.15E-03	1.15E-03	1.15E-03
P_0 (cm/s)	1.74E-04	1.74E-04	1.74E-04
x_a (cm)	0.0133	0.0133	0.0133
x_b (cm)	0.0293	0.0293	0.0293
D_w (cm^2/s)	6.91E-06	6.91E-06	6.91E-06
pK_a	9.4	9.4	9.4
S	24	24	24

Table S8: Parameter table for **Doxorubicin.**

Fit Parameters	**A:** Neutral-Neutral	**B:** Ionic-Ionic	**C:** Neutral-Ionic
$P_{pgp,app}^{active}$ (cm/s)	**3.41E-01**	**6.74E-03**	**2.21E-01**
$P_{b,app}^{active}$ (cm/s)	**1.00E-04**	**2.46E-07**	**2.46E-07**
P_{para} (cm/s)	**1.60E-07**	**1.56E-07**	**1.57E-07**
P_{filter} (cm/s)	6.28E-04	6.28E-04	6.28E-04
P_{cyt} (cm/s)	9.58E-04	9.58E-04	9.58E-04
P_0 (cm/s)	6.23E-05	6.23E-05	6.23E-05
x_a (cm)	0.0133	0.0133	0.0133
x_b (cm)	0.0293	0.0293	0.0293
D_w (cm^2/s)	5.75E-06	5.75E-06	5.75E-06
pK_a	9.56	9.56	9.56
S	24	24	24

Table S9: Parameter table for **Etoposide.**

Fit Parameters	**A:** Neutral-Neutral	**B:** Ionic-Ionic	**C:** Neutral-Ionic
$P_{pgp,app}^{active}$ (cm/s)	**1.72E-03**	**1.00E-01[a]**	**1.00E-01[a]**
$P_{b,app}^{active}$ (cm/s)	**1.36E-06**	**1.34E-07**	**1.23E-07**
P_{para} (cm/s)	**9.36E-08**	**1.37E-07**	**1.45E-07**
P_{filter} (cm/s)	6.06E-04	6.06E-04	6.06E-04
P_{cyt} (cm/s)	9.24E-04	9.24E-04	9.24E-04
P_0 (cm/s)	8.27-06	8.27-06	8.27-06
x_a (cm)	0.0133	0.0133	0.0133
x_b (cm)	0.0293	0.0293	0.0293
D_w (cm^2/s)	5.55E-06	5.55E-06	5.55E-06
pK_a	8.53	8.53	8.53
S	24	24	24

[a] *Constrained to be less than 1E-01 cm/s*

Table S10: Parameter table for **Digoxin.**

Fit Parameters	**A:** Neutral-Neutral	**B:** Ionic-Ionic	**C:** Neutral-Ionic
$P_{pgp,app}^{active}$ (cm/s)	**2.64E-03**	**n/A**	**3.76E-03**
$P_{b,app}^{active}$ (cm/s)	**2.12E-06**	**n/A**	**1.00E-09**
P_{para} (cm/s)	**1.52E-07**	**n/A**	**2.0E-07[a]**
P_{filter} (cm/s)	5.33E-04	5.33E-04	5.33E-04
P_{cyt} (cm/s)	8.13E-04	8.13E-04	8.13E-04
P_0 (cm/s)	2.69-06	2.69-06	2.69-06
x_a (cm)	0.0133	0.0133	0.0133
x_b (cm)	0.0293	0.0293	0.0293
D_w (cm^2/s)	4.88E-06	4.88E-06	4.88E-06
S	24	24	24

[a] *Held at 2E-07 cm/s*

Table S11: Parameter table for **Colchicine.**

Fit Parameters	**A:** Neutral-Neutral	**B:** Ionic-Ionic	**C:** Neutral-Ionic
$P_{pgp,app}^{active}$ (cm/s)	**5.95E-03**	**8.68E+04**	**1.33E-02**
$P_{b,app}^{active}$ (cm/s)	**2.03E-08**	**3.96E-01**	**0.10E-01**
P_{para} (cm/s)	**2.0E-07[a]**	**2.0E-07[a]**	**2.16E-07**
P_{filter} (cm/s)	7.24E-04	7.24E-04	7.24E-04
P_{cyt} (cm/s)	1.10E-03	1.10E-03	1.10E-03
P_0 (cm/s)	1.25E-06	1.25E-06	1.25E-06
x_a (cm)	0.0133	0.0133	0.0133
x_b (cm)	0.0293	0.0293	0.0293
D_w (cm^2/s)	6.63E-06	6.63E-06	6.63E-06
S	24	24	24

[a] *Held at 2E-07 cm/s*

VIII General model curves

In Section 4.1 of the main text, general curves for a basic compound were shown under the neutral-neutral assumption to visually demonstrate the pH dependence of the P_{app} in both directions, and accompanying ER with and without the influence of paracellular transport and the basolateral influx transporter. These curves were generated using Igor Pro 7 for a theoretical basic substrate with very general properties: a molecular weight of 340 g/mol, a basic pKa of 9.2 and a P_0 of 4.2 x 10^{-5} cm/s. The curves were produced by a stepwise increase in complexity. In scenario **A**, we simulate the situation where only P-gp is present, with no paracellular transport and no basolateral influx. To assess the influence of P-gp, we perform a sensitivity analysis where P_{pgp} is set to 1 x, 5 x, 10 x, 50 x and 100 x passive diffusion. In scenario **B**, we add paracellular transport with a plausible value of 1.2 x 10^{-7} cm/s. In scenario **C**, we further added basolateral influx transport. Figures S7-S10 shows the resultant P_{app} and ER values as a function of pH for each of the scenarios, under each of the transporter preference assumptions investigated in the main text (1: neutral-neutral, 2: ionic-ionic, 3: neutral-ionic).

For all graphs, the left panel shows the P_{app} in cm/s, with $P_{app,A\text{-}B}$ (in varying shades of blue depending on the magnitude of P_{pgp}) and $P_{app,B\text{-}A}$ in red. The right panel shows the ER as a function of pH, also with different curves based on the magnitude difference between P_{gpp} and passive diffusion. For the neutral-neutral assumption (1, Figure S7), scenarios A and C are presented in the main text and therefore omitted here. For the neutral-ionic assumption (4, Figure S9), scenarios A and B are omitted, as the absence of basolateral influx in these scenarios would lead to the same results as under the neutral-neutral assumption already presented. From these curves a few general conclusions can be easily visualised for a better understanding. For one, it can be seen that $P_{app,B\text{-}A}$ is unaffected by changes in the magnitude of P_{pgp}, unlike $P_{app,A\text{-}B}$ which is very much dependent on it. The absence of paracellular transport in the **A** scenarios means that the unavoidable lower plateau is never captured, resulting in irrationally high efflux ratios at low pH values that would never be achieved experimentally. The introduction of paracellular transport in the **B** scenarios immediately tempers this effect and results in substantially lower ERs. Introducing paracellular transport also enables the visualisation of how its dominance at low pH values (i.e at low f_n for this basic compound) reduces the ER to unity. The introduction of basolateral influx in the **C** scenarios captures the substantially higher $P_{app,B\text{-}A}$ values also observed experimentally, and which P-gp activity alone can simply not describe. Basolateral influx also moderately increases the ER. For the ionic-ionic (Figure S8) assumption, the drop in ER that was discussed as a potential indicator of (at least partial) ion transport in the main text can also be observed, as with the neutral-neutral assumption (Figure S7), the ER plateaus at higher pH values instead.

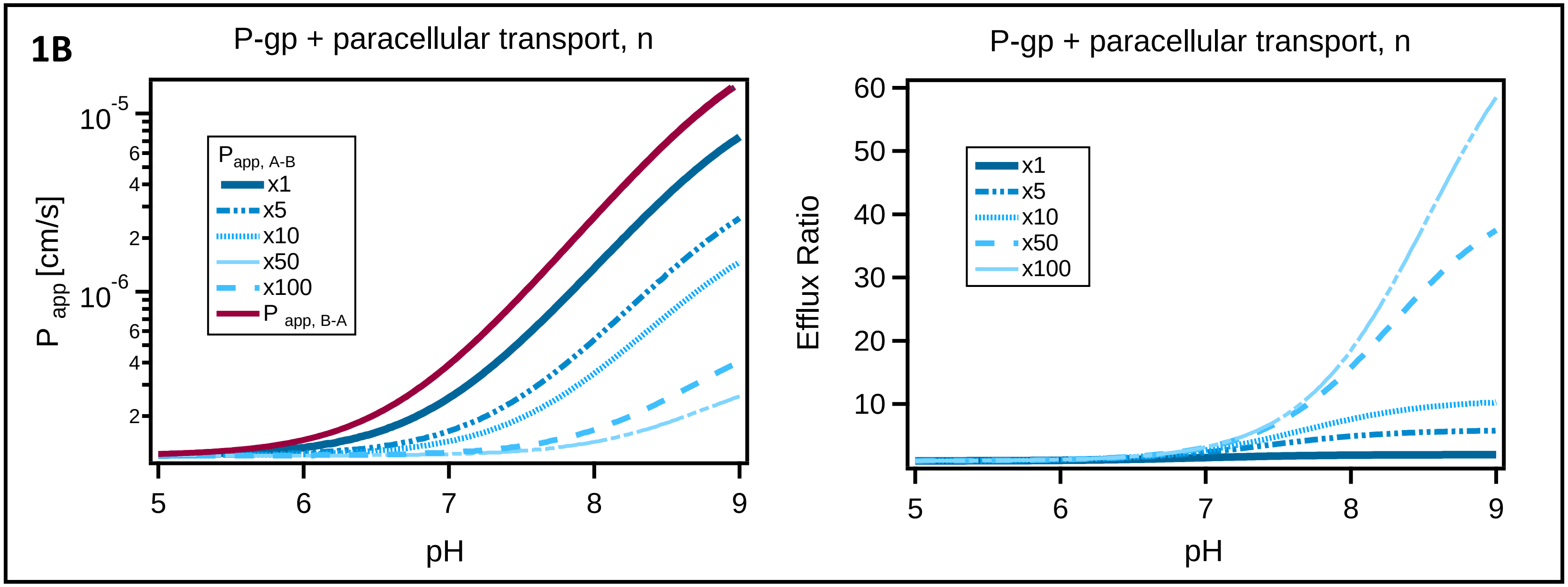

Figure S7: Apparent permeability (left) and efflux ratio (right) as a function of pH for a basic compound under assumption that both transporters act on the neutral species: B) P-gp efflux and paracellular transport. Scenarios A and C under the neutral-neutral assumption are presented in the main text. $P_{app,A\rightarrow B}$ (left panel, blue) changes according to magnitude of P_{pgp}. Sensitivity analysis curves depict $P_{app,A\rightarrow B}$ and ER when P_{pgp} is varied by increments of 1x, 5x *etc* passive diffusion. $P_{app,B\rightarrow A}$ (left panel, red) is independent of changes in P_{pgp}.

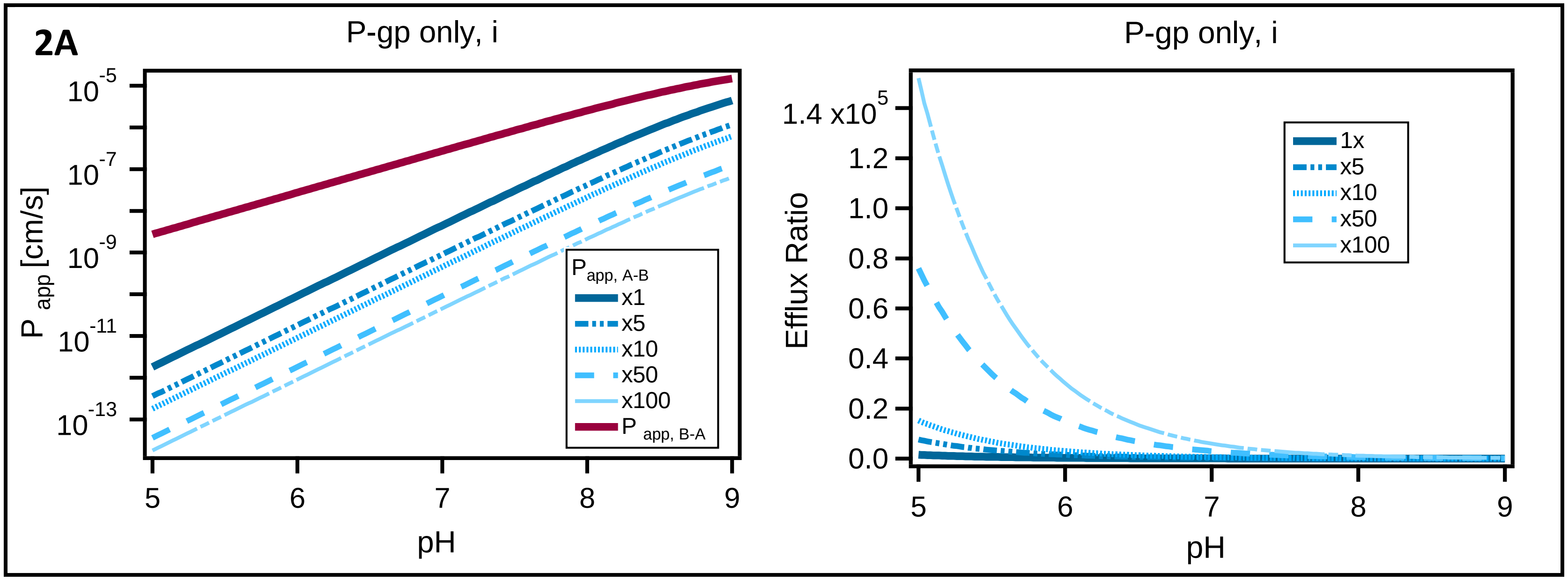

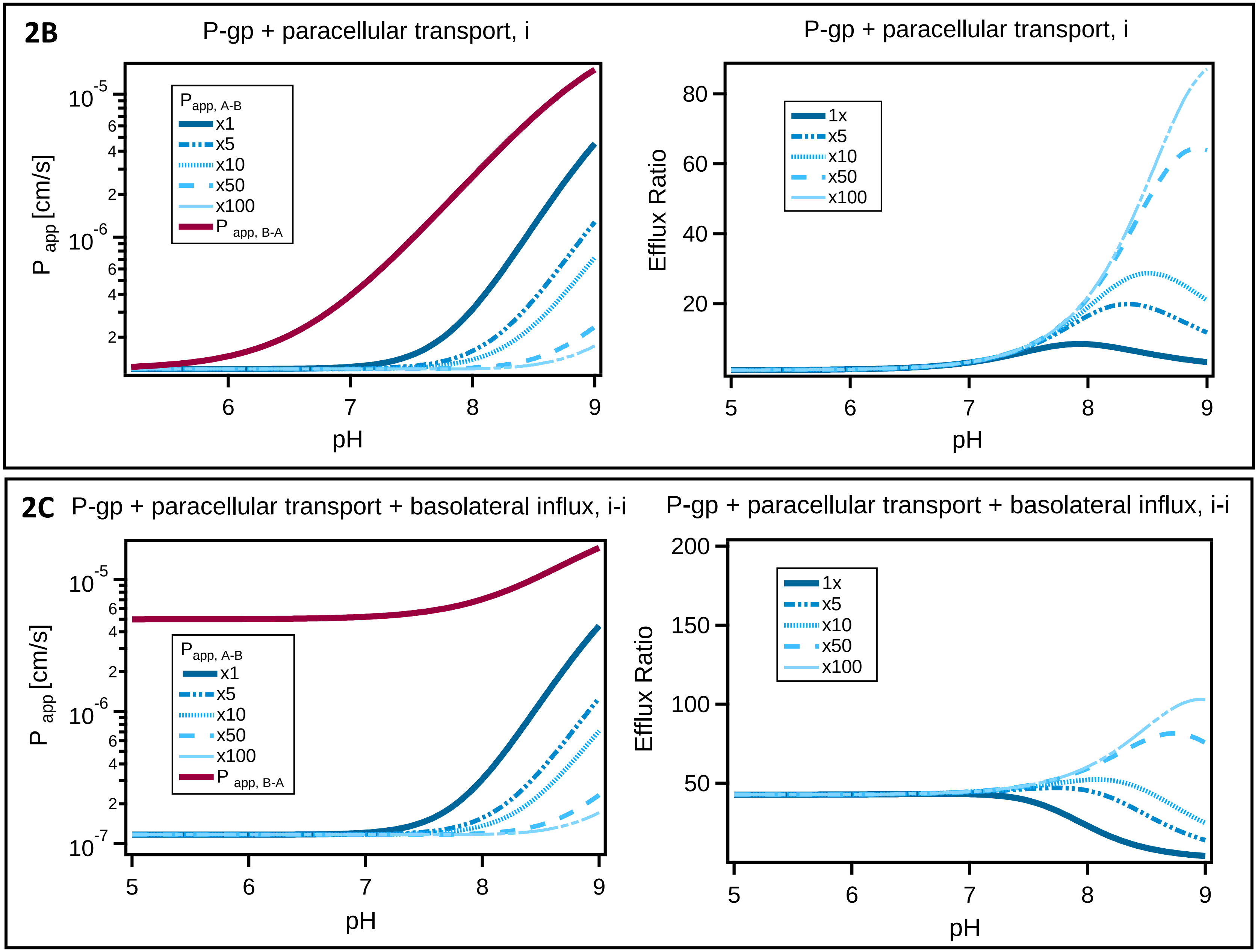

Figure S8: Apparent permeability and efflux ratio as a function of pH for a basic compound under the assumption that both transporters prefer the ionic species: A) with P-gp efflux only, B) P-gp efflux and paracellular transport and C) P-gp efflux, paracellular transport and basolateral influx transport. $P_{app,A\rightarrow B}$ (left panel, blue) changes according to magnitude of P_{pgp}. Sensitivity analysis curves depict $P_{app,A\rightarrow B}$ and ER when P_{pgp} is varied by increments of 1x, 5x *etc* passive diffusion. $P_{app,B\rightarrow A}$ (left panel, red) is independent of changes in P_{pgp}.

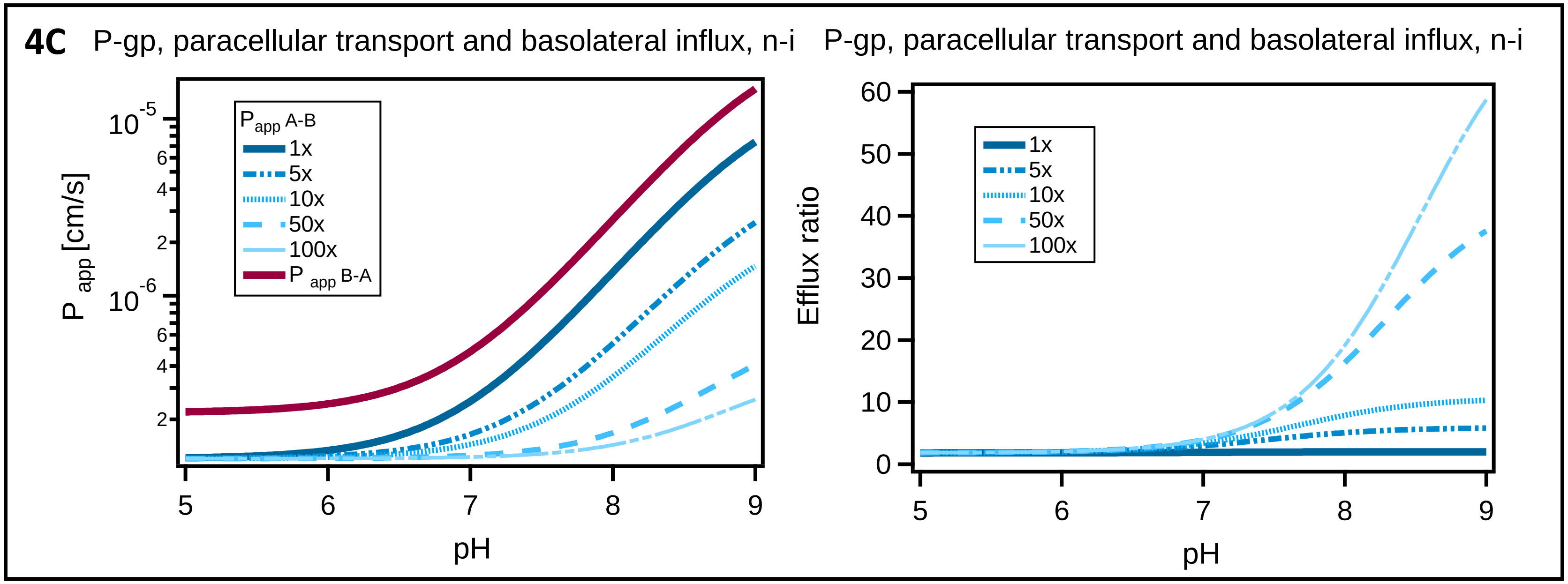

Figure S9: Apparent permeability and efflux ratio as a function of pH for a basic compound under assumption that P-gp refers the neutral species for transport, and the basolateral transporter prefers the ionic species: C) P-gp efflux, paracellular transport and basolateral influx transport. Scenarios A and B is equivalent to 1A and 1C, and are therefore not presented uniquely. $P_{app,A\rightarrow B}$ (left panel, blue) changes according to magnitude of P_{pgp}. Sensitivity analysis curves depict $P_{app,A\rightarrow B}$ and ER when P_{pgp} is varied by increments of 1x, 5x *etc* passive diffusion. $P_{app,B\rightarrow A}$ (left panel, red) is independent of changes in P_{pgp}.

IX Model fits and parameters of pH-gradient data

Fits were also performed for the pH-gradient data generated by Neuhoff *et al*. (2003) in order to see whether any additional information could be extracted from such data. Similar to our own experiments, we generated these fits under all three assumptions for the compound talinolol, as well as under the neutral-neutral assumption for the non-dissociating compound digoxin. Figures S5 and S6 show the results of these fits with the accompanying parameter tables. The individual ABL thicknesses (x_a and x_b) have been determined previously for the Caco-2 system of Neuhoff by Dahley *et al*. (2023). P_{filter} has also been adjusted to account for the different filter used. Once again, for these parameter tables the first three rows show the parameters left free to be fitted in bold, while the rest of the parameters were fixed at the given values. As stated in the main text, fitting these pH-gradient data did generate some interesting conclusions. As with our own data generated with the iso-pH method, the pH-gradient data can only be explained with the inclusion of a basolateral transporter, since the high $P_{app,\ B\rightarrow A}$ obtained experimentally could not be achieved without P_b^{active}. However, because of the pH-gradient, P_b^{active} does not vary across the entire pH range, which confounds any information or conclusions that might otherwise have been interesting regarding this transport. For the non-dissociating compound digoxin (Figure S10), the model was able to produce a reasonable fit. One can see that the P_b^{active} is one order of magnitude higher than that obtained for our MDCK results with the iso-pH method for the same compound, while P_{pgp}^{active} is two orders of magnitude lower.

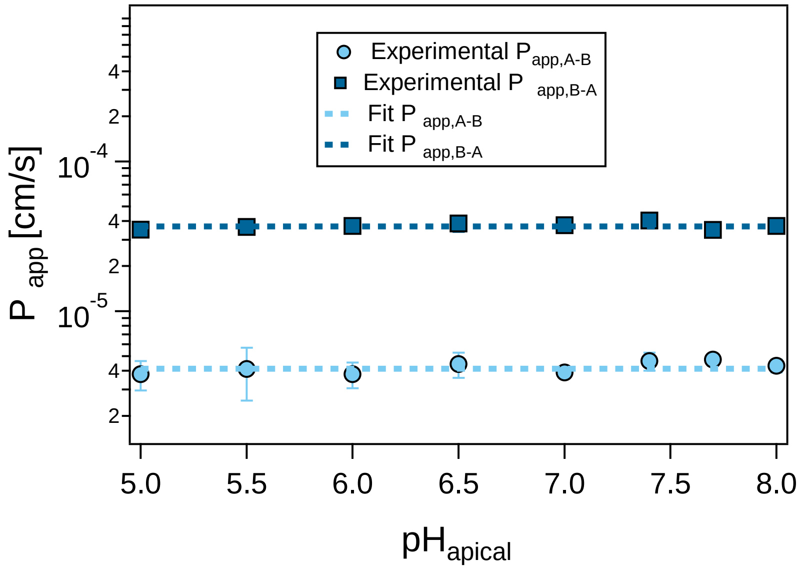

Parameters	
**$P_{pgp,app}^{active}$ (cm/s)**	**6.39E-05**
**$P_{b,app}^{active}$ (cm/s)**	**5.16E-05**
**P_{para} (cm/s)**	**5.22E-06**
P_{filter} (cm/s)	6.13E-04
P_{cyt} (cm/s)	8.13E-04
P_0 (cm/s)	2.69-06
x_a (cm)	0.0159
x_b (cm)	0.0391
D_w (cm²/s)	4.88E-06
S	24

Figure S10: Experimental apparent permeability as a function of apical compartment pH for digoxin (Neuhoff et al. 2003) plotted with model fits, and the accompanying parameter table. Basolateral pH was always 7.4. Experimental $P_{app,A\rightarrow B}$ (light blue markers) and $P_{app,B\rightarrow A}$ (dark blue markers) represent the mean of at least three replicates ($n \geq 3$). Error bars represent standard deviation. The fits were performed using Igor Pro 7 and are shown as dashed lines. All P_{app} values were included in the fit and weighted by standard deviation. Fits were performed under the assumption that both transporters act on the neutral fraction.

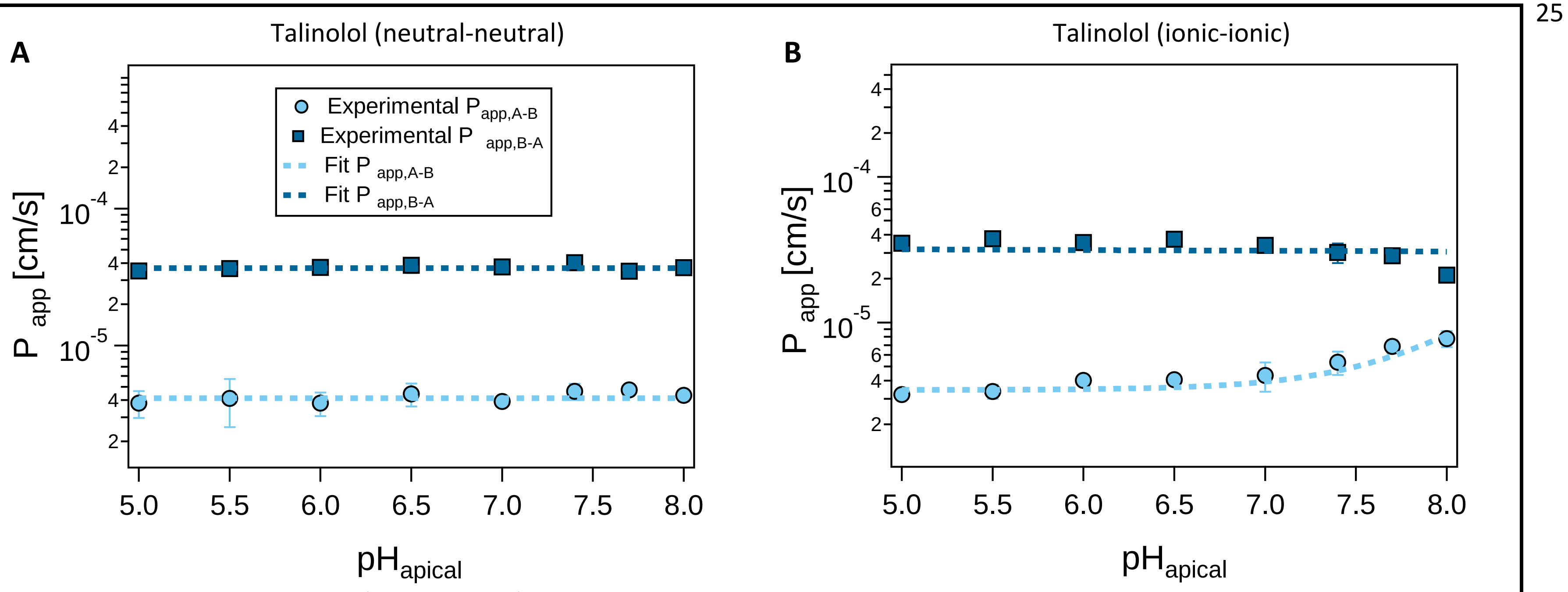

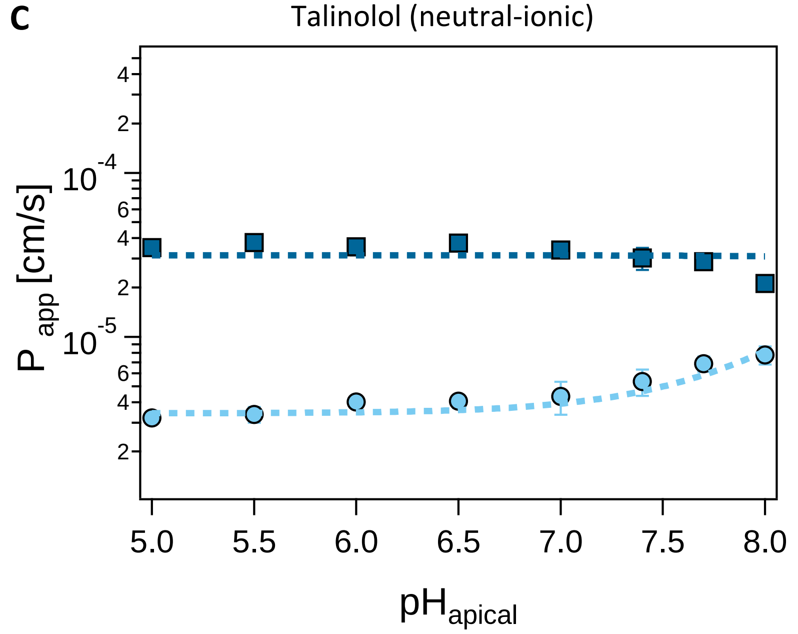

Figure S11: Experimental apparent permeability as a function of apical compartment pH for talinolol (Neuhoff et al. 2003) plotted along with model fits under varying assumptions. The basolateral pH was always 7.4 Experimental $P_{app,A\rightarrow B}$ (light blue markers) and $P_{app,B\rightarrow A}$ (dark blue markers) represent the mean of at least three replicates ($n \geq 3$). Error bars represent standard deviation. The fits were performed using Igor Pro 7 and are shown as dashed lines. All P_{app} values were included in the fit and weighted by standard deviation. Fits were performed under three different assumptions: A) Both transporters act on the neutral fraction, B) Both transporters act on the ionic fraction and C) The apical transporter acts on the neutral species, and the basolateral transporter acts on the ionic species.

Table S12: Parameter table for Talinolol gradient pH method *(Neuhoff et al. 2003)*.

Fit Parameters	**A:** Neutral-Neutral	**B:** Ionic-Ionic	**C:** Neutral-Ionic
$\boldsymbol{P^{active}_{pgp,app}}$ (cm/s)	**1.00E-04**[a]	**5.21E-06**	**1.00E-04**[a]
$\boldsymbol{P^{active}_{b,app}}$ (cm/s)	**3.58E-03**	**3.55E-05**	**5.62E-05**
$\boldsymbol{P_{para}}$ (cm/s)	**4.42E-06**	**4.44E-06**	**4.40E-06**
P_{filter} (cm/s)	8.69E-04	8.69E-04	8.69E-04
P_{cyt} (cm/s)	1.15E-03	1.15E-03	1.15E-03
P_0 (cm/s)	1.74E-04	1.74E-04	1.74E-04
x_a (cm)	0.0159	0.0159	0.0159
x_b (cm)	0.0391	0.0391	0.0391
D_w (cm^2/s)	6.91E-06	6.91E-06	6.91E-06
pK_a	9.4	9.4	9.4
S	24	24	24

[a] *Upper limit value*

Likewise, for the basic compound talinolol, the graphs in Figure S11 show that the model was also able to fit the data well. Table S12 shows the parameters for these fits. Under the neutral-neutral and neutral-ionic assumptions, P_{pgp}^{active} seems to not play any significant role, as it is consistently fitted as a negative value. However, when P_{pgp}^{active} is fixed to be positive at higher values, this does not meaningfully change the fit until some maximum value is reached, whereafter $P_{app, A\rightarrow B}$ starts to drop at the high end of the pH range. Therefore, fitting these data under these two assumptions seems to at most provide an upper limit for P_{pgp}^{active}, as indicated in the Table S12. This is not altogether trivial, as it does lead to the interesting insight that even the maximum value for P_{pgp}^{active} in the Caco-2 cells is 10 times less than the P_{pgp}^{active} value obtained in our MDCK experiments, which could be an interesting quasi-quantification of the expected difference of P-gp activity between these two cell lines. The increased P_{pgp}^{active} in MDCK-MDR1 cells is a predictable consequence of greater P-gp expression in these transfected cells. Additionally, the difference in surface area between the two cell lines could also partially contribute to the observed increase.

X Measurement of paracellular transport

With our work we have stressed the importance of accounting for paracellular transport. In order to assess its relative contribution to overall flux, it is necessary to quantify paracellular transport in some way. An approximation of paracellular transport can be mathematically calculated as described in Bitterman and Goss (2017) and Avdeef (2010), yet many factors, such as molecule size, molecule charge and cell type can influence and complicate the predictions, leading to uncertainties. However, it can also be experimentally measured provided that it can be assured that the P_{app} would be dominated by the contribution of P_{para}. Table S13 depicts the P_{app} values obtained for two acidic and two basic compounds. In order to ensure paracellular dominance, 2 µM elacridar was used to inhibit potential P-gp facilitated efflux, and the transport buffer was set to a pH that would severely restrict the neutral fraction available. For the MDCK-MDR1 cells, the results showed that basic compounds tend to have a lower $\log P_{para}$ than acidic compounds.

Table S13: P_{app} values and measured $logP_{para}$ for basic and acidic marker compounds. P_{app} values are the mean of three timepoints per replicate, for a total of three replicates (n = 3) ± standard deviation.

Compound		pH	$P_{app, A \rightarrow B}$ [x 10^{-6} cm/s]	$logP_{para}$	Recovery [%]	f_n [%]
Bases	Doxorubicin + elacridar	5	0.4 ± 0.1	-6.4	130	0.0
	Acebutolol + elacridar	5	0.6 ± 0.2	-6.2	100	0.0
Acids	Chlorothiazide + elacridar	9	1.7 ± 0.7	-5.8	105	0.8
	Furosemide + elacridar	9	1.5 ± 0.4	-5.8	100	0.0

In light of the uncertainties in predicting P_{para}, it was presumed more apt to measure P_{para} for each compound individually in parallel with the pH dependence experiments. As described in Section 4.3 of the main text, experiments were initially performed for each tested compound with inhibitor and appropriate pH values (similar to those performed for Table S12) in an attempt to ensure the dominance of paracellular transport. These values could then be used as a direct measure for P_{para} for each compound that could be fed to the model for its individual fit. However, these experimentally-derived values were consistently in the range of 1E-05 to 1E-06, values for P_{para} that were simply too high for the model to be able to fit the data well. Under the suspicion that the use of inhibitor may be affecting P_{para} and/or the measurement of it, we measured the P_{app} of more commonly-used markers for paracellular transport with and without inhibitor. The transport of these rather hydrophilic compounds is known to be dominated by the paracellular route. Furthermore, for our MDCK-MDR1 cells, these compounds did not have a significant ER when measured without inhibitor, and therefore efflux is not expected to play a role. As such, the addition of inhibitor in this case is purely to evaluate if the P_{app} changes when it is present. Table S14 shows the results for the compounds atenolol and furosemide.

Table S14: P_{app} values and calculated $logP_{para}$ for marker compounds with and without 2 µM elacridar. P_{app} values are the mean of three timepoints per replicate, for one replicate ± standard deviation.

Compound	pH	P_{app} [x 10^{-6} cm/s]	$logP_{para}$	Recovery [%]	f_n [%]	ER
Atenolol A⟶ B	6	0.1 ± 0.0	-6.9	100	0.1	0.8
Atenolol B⟶ A	6	0.1 ± 0.0	-7.0	107	0.1	
Atenolol A⟶ B + elacridar	6	0.4 ± 0.0	-6.4	100	0.1	-
Furosemide A⟶ B	9	0.4 ± 0.1	-6.4	99	0.0	1.0
Furosemide B⟶ A	9	0.4 ± 0.1	-6.4	100	0.0	
Furosemide A⟶ B + elacridar	9	1.1 ± 0.4	-6.0	101	0.0	-

As is evident from these data, the addition of the inhibitor elacridar tends to increase the P_{app} and thus the $logP_{para}$ derived from it by ca. 0.5 log units. In order to assess whether this effect was a consequence of specifically using elacridar as an inhibitor, we evaluated whether other known P-gp inhibitors also change the measured P_{para}. Table S15 (an expansion of Table 1 in the main text, with additional information) compares the results obtained for the attempted measurement of P_{para} for the non-P-gp

substrate chlorothiazide without inhibitor, and with three different P-gp inhibitors: elacridar (2 µM), verapamil (100 µM) and cyclosporin A (10 µM).

Table S15: Comparison of P_{app} values and calculated $logP_{para}$ for chlorothiazide with various P-gp inhibitors. P_{app} values are the mean of three timepoints per replicate, for one replicate ± standard deviation.

Compound	pH	P_{app} [x 10^{-6} cm/s]	$logP_{para}$	Recovery [%]	f_n [%]	ER
Chlorothiazide A⟶ B	7.4	0.2 ± 0.0	-6.6	125	16.5	1.0
Chlorothiazide B⟶ A	7.4	0.2 ± 0.0	-6.7	104	16.5	
Chlorothiazide A⟶ B + elacridar	7.4	0.9 ± 0.0	-6.0	100	16.5	-
Chlorothiazide A⟶ B + verapamil	7.4	1.4 ± 0.1	-5.9	96	16.5	-
Chlorothiazide A⟶ B + cyclosporin-A	7.4	0.7 ± 0.0	-6.1	108	16.5	-

From Table S15 it is clear that it is not only elacridar that tends to affect measurement of P_{para}. For all three inhibitors the measured $logP_{para}$ was higher with its addition to the transwell assay. It was decided that we could not obtain reliable measurement for P_{para} using our initial method (i.e reducing the neutral fraction and using inhibitor). The inability of the model to fit the data when P_{para} was fixed at the values in the range of 1E-05 to 1E-06 that were obtained with such experiments led us to probe these measurements, and the data presented in Tables S13-S15 casts doubt on them. It is plausible that the use of inhibitor in these assays either affects P_{para} or its measurement. This conclusion led to the decision to leave P_{para} free to be fitted by the model, and it was rather consistently estimated to be within the range of 1-5 x 10^{-7} cm/s (see Tables S6-S11), which translates to a $logP_{para}$ of between -7 and -6.3. These values correspond rather more favourably with the $logP_{para}$ obtained above for non-P-gp substrate compounds measured without inhibitor, whose transport is expected to be dominated by the paracellular route.

References

Avdeef, A. (2010). Leakiness and size exclusion of paracellular channels in cultured epithelial cell monolayers-interlaboratory comparison. *Pharmaceutical Research*, *27*(3), 480–489. https://doi.org/10.1007/s11095-009-0036-7

Avdeef, A. Absorption and Drug Development: Solubility, Permeability, and Charge State, 2nd ed.; *John Wiley & Sons*, Inc.: Hoboken, NJ, USA, 2012

Bittermann, K., & Goss, K. U. (2017). Predicting apparent passive permeability of Caco-2 and MDCK cell-monolayers: A mechanistic model. *PLoS ONE*, *12*(12). https://doi.org/10.1371/journal.pone.0190319

Callegari, E., Malhotra, B., Bungay, P. J., Webster, R., Fenner, K. S., Kempshall, S., Laperle, J. L., Michel, M. C., & Kay, G. G. (2011). A comprehensive non-clinical evaluation of the CNS penetration potential of antimuscarinic agents for the treatment of overactive bladder. *British Journal of Clinical Pharmacology*, *72*(2), 235–246. https://doi.org/10.1111/j.1365-2125.2011.03961.x

Chang, C., Bahadduri, P. M., Polli, J. E., Swaan, P. W., & Ekins, S. (2006). Rapid identification of P-glycoprotein substrates and inhibitors. *Drug Metabolism and Disposition*, *34*(12), 1976–1984. https://doi.org/10.1124/dmd.106.012351

Dahley, C., Goss, K. U., & Ebert, A. (2023). Revisiting the pKa-Flux method for determining intrinsic membrane permeability. *European Journal of Pharmaceutical Sciences*, *191*. https://doi.org/10.1016/j.ejps.2023.106592

de Souza, J., Benet, L. Z., Huang, Y., & Storpirtis, S. (2009). Comparison of bidirectional lamivudine and zidovudine transport using MDCK, MDCK-MDR1, and Caco-2 cell monolayers. *Journal of Pharmaceutical Sciences*, *98*(11), 4413–4419. https://doi.org/10.1002/jps.21744

Eriksson, U. G., Dorani, H., Karlsson, J., Fritsch, H., Hoffmann, K. J., Olsson, L., Sarich, T. C., Wall, U., & Schützer, K. M. (2006). Influence of erythromycin on the pharmacokinetics of ximelagatran may involve inhibition of P-glycoprotein-mediated excretion. *Drug Metabolism and Disposition*, *34*(5), 775–782. https://doi.org/10.1124/dmd.105.008607

Escher, B. I., Abagyan, R., Embry, M., Klüver, N., Redman, A. D., Zarfl, C., & Parkerton, T. F. (2020). Recommendations for Improving Methods and Models for Aquatic Hazard Assessment of Ionizable Organic Chemicals. *Environmental Toxicology and Chemistry* (Vol. 39, Issue 2, pp. 269–286). Wiley Blackwell. https://doi.org/10.1002/etc.4602

Gertz, M., Harrison, A., Houston, J. B., & Galetin, A. (2010). Prediction of human intestinal first-pass metabolism of 25 CYP3A substrates from in vitro clearance and permeability data. *Drug Metabolism and Disposition*, *38*(7), 1147–1158. https://doi.org/10.1124/dmd.110.032649

Hasselbalch, K. A. *Die Berechnung Der Wasserstoffzahl Des Blutes Aus Der Freien Und Gebundenen KohlensäUre Desselben, Und Die Sauerstoffbindung Des Blutes Als Funktion Der Wasserstoffzahl*; Julius Springer: Berlin, 1916.

Henderson LJ. The regulation of neutrality in the human body. *Science*. 1913 Mar 14;37(950):389-95. doi: 10.1126/science.37.950.389. PMID: 17795147.

Karlsson, J.; Artursson, P. A method for the determination of cellular permeability coefficients and aqueous boundary layer thickness in monolayers of intestinal epithelial (Caco-2) cells grown in permeable filter chambers. *Int. J. Pharm*. 1991, 71, 55–64

Liu, W., Okochi, H., Benet, L. Z., & Zhai, S. di. (2012). Sotalol permeability in cultured-cell, rat intestine, and PAMPA system. *Pharmaceutical Research*, *29*(7), 1768–1774. https://doi.org/10.1007/s11095-012-0699-3

Mahar Doan, K. M., Humphreys, J. E., Webster, L. O., Wring, S. A., Shampine, L. J., Serabjit-Singh, C. J., Adkison, K. K., & Polli, J. W. (2002). Passive permeability and P-glycoprotein-mediated efflux differentiate central nervous system (CNS) and non-CNS marketed drugs. *Journal of Pharmacology and Experimental Therapeutics*, *303*(3), 1029–1037. https://doi.org/10.1124/jpet.102.039255

S. Neuhoff, A.-L. Ungell, I. Zamora, P. Artursson, ph-dependent bidirectional transport of weakly basic drugs across caco-2 monolayers: Implications for drug-drug interactions, *Pharmaceutical Research,* 20 (2003) 1141–1147.

Obradovic, T., Dobson, G. G., Shingaki, T., Kungu, T., & Hidalgo, I. J. (2007). Assessment of the first and second generation antihistamines brain penetration and role of P-glycoprotein. *Pharmaceutical Research*, *24*(2), 318–327. https://doi.org/10.1007/s11095-006-9149-4

Palay, S.L.; Karlin, L.J. An Electron Microscopic Study of the Intestinal Villus: II. The Pathway of Fat Absorption. *J. Biophys. Biochem. Cytol.* 1959, 5, 373–384.

Polli, J. W., Wring, S. A., Humphreys, J. E., Huang, L., Morgan, J. B., Webster, L. O., & Serabjit-Singh, C. S. (2001). Rational Use of in Vitro P-glycoprotein Assays in Drug Discovery.

Tang, F., Ouyang, H., Yang, J. Z., & Borchardt, R. T. (2004). Bidirectional Transport of Rhodamine 123 and Hoechst 33342, Fluorescence Probes of the Binding Sites on P-glycoprotein, across MDCK-MDR1 Cell Monolayers. *Journal of Pharmaceutical Sciences*, (Vol. 93, Issue 5).

Taub, M. E., Podila, L., Ely, D., & Almeida, I. (2005). Functional assessment of multiple p-glycoprotein (P-gp) probe substrates: influence of cell line and modulator concentration on P-gp activity. *Drug Metabolism and Disposition*, *33*(11), 1679–1687. https://doi.org/10.1124/dmd.105.005421

Troutman, M. D., & Thakker, D. R. (2003). Novel Experimental Parameters to Quantify the Modulation of Absorptive and Secretory Transport of Compounds by P-Glycoprotein in Cell Culture Models of Intestinal Epithelium.

Verkman AS. Solute and macromolecule diffusion in cellular aqueous compartments. *Trends Biochem Sci*. 2002 Jan;27(1):27-33. doi: 10.1016/s0968-0004(01)02003-5. PMID: 11796221.

Wang, Q., Rager, J. D., Weinstein, K., Kardos, P. S., Dobson, G. L., Li, J., & Hidalgo, I. J. (2005). Evaluation of the MDR-MDCK cell line as a permeability screen for the blood-brain barrier. *International Journal of Pharmaceutics*, *288*(2), 349–359. https://doi.org/10.1016/j.ijpharm.2004.10.007
